# Multi-platform integration of brain and CSF proteomes reveals biomarker panels for Alzheimer’s disease

**DOI:** 10.1093/bib/bbag012

**Published:** 2026-01-29

**Authors:** Wei-Yun Tsai, Pieter Giesbertz, Stephan Breimann, Stefan Lichtenthaler, Dmitrij Frishman

**Affiliations:** Department of Bioinformatics, School of Life Sciences, Technical University of Munich, Maximus‑von‑Imhof‑Forum 3, 85354 Freising, Germany; German Center for Neurodegenerative Diseases, Feodor-Lynen-Street 17, 81377 Munich, Germany; Neuroproteomics, School of Medicine and Health, Technical University of Munich University Hospital, Technical University of Munich, Ismaninger Str. 22, 81675 Munich, Germany; Department of Bioinformatics, School of Life Sciences, Technical University of Munich, Maximus‑von‑Imhof‑Forum 3, 85354 Freising, Germany; German Center for Neurodegenerative Diseases, Feodor-Lynen-Street 17, 81377 Munich, Germany; Neuroproteomics, School of Medicine and Health, Technical University of Munich University Hospital, Technical University of Munich, Ismaninger Str. 22, 81675 Munich, Germany; German Center for Neurodegenerative Diseases, Feodor-Lynen-Street 17, 81377 Munich, Germany; Neuroproteomics, School of Medicine and Health, Technical University of Munich University Hospital, Technical University of Munich, Ismaninger Str. 22, 81675 Munich, Germany; Munich Cluster for System Neurology, Feodor-Lynen-Street 17, 81377 Munich, Germany; Department of Bioinformatics, School of Life Sciences, Technical University of Munich, Maximus‑von‑Imhof‑Forum 3, 85354 Freising, Germany

**Keywords:** data integration, machine learning, proteomics, biomarkers, CSF, brain tissue

## Abstract

Alzheimer’s disease (AD) is the leading cause of dementia and represents a progressive, irreversible neurodegenerative disorder. Given the complexity and heterogeneity of AD, which involves numerous interrelated molecular pathways, large-scale proteomics datasets are essential for robust biomarker discovery. Comprehensive proteomic profiling enables the unbiased identification of novel biomarkers across diverse biological processes, thereby increasing the likelihood of finding sensitive and specific candidates for early diagnosis and therapeutic targeting. In this study, we analyzed 28 large-scale proteomics datasets obtained from the AD Knowledge Portal and published studies. The data comprise tandem mass tag, label-free quantification, and proximity extension assay measurements from brain tissue and cerebrospinal fluid. To enhance analytical power, we integrated these proteomic profiles with corresponding clinical information to construct comprehensive feature sets for subsequent machine learning analysis. Using Random Forest and Logistic Regression models, we identified a panel of proteins capable of distinguishing AD patients from healthy controls. Several of these biomarkers have been previously validated in the context of AD, while others represent novel candidates not yet reported as AD-associated. These newly identified biomarkers warrant further experimental validation and hold promise for improving early diagnosis as well as guiding the development of targeted therapies for AD.

## Introduction

Alzheimer’s disease (AD) is a progressive neurodegenerative disorder and the most common cause of dementia, characterized by cognitive decline, memory loss, and behavioral changes [1]. AD affects ~50 million lives worldwide according to the World Health Organization, and this number is expected to dramatically increase in the near future, reaching 152 million in 2050 [2]. One of the key challenges in addressing Alzheimer’s lies in the difficulty of early and accurate diagnosis, as well as in the monitoring of disease progression and treatment response.

In AD, specific proteins such as amyloid-beta (Aβ) peptides, tau, and phosphorylated tau have been linked to hallmark pathological features such as amyloid plaques and neurofibrillary tangles [3]. These proteins, detectable in cerebrospinal fluid (CSF) and increasingly in blood-based assays, provide insights into disease onset and progression long before the appearance of clinical symptoms [4]. Yet, AD is a systemic disorder and is assumed to affect all cell types in the brain, raising the possibility that many more proteins may be suitable biomarkers for AD pathogenesis.

An increasing amount of protein abundance data from AD patients (from brain tissue or body fluids, such as plasma and CSF) has become publicly available and may offer the identification and validation of additional protein biomarkers. While traditional computational approaches (e.g. one-way ANOVA or hierarchical clustering) led to the identification of AD biomarkers within these datasets, it has been difficult to use such standard computational tools in an overarching way to merge and consider data from diverse sources. For example, these studies typically analyzed whether single proteins correlated with disease but did not attempt to test whether a panel of proteins and additional clinical features may provide a much better disease marker or allow a stratification of AD patients into different subgroups. Thus, AD research would strongly benefit from machine learning-based data analysis by combining multiple datasets from different cohorts. While this approach has been widely used for transcriptomics and genetic data [5], and some studies [6, 7] have applied machine learning to Alzheimer’s proteomics data, such analyses remain challenging because of missing values, the lower number of proteins identified compared to transcripts, and the different technical data acquisition methods employed. Therefore, the development of sophisticated machine learning models is required for realizing the full potential of large-scale proteome data in AD studies [8, 9].

In this study we used machine learning to integrate proteomic data from brain tissue and CSF along with clinical information. By addressing challenges such as missing data and variability across experiments, we aimed at identifying biomarkers that can distinguish AD patients from healthy controls.

## Materials and methods

### Overview of the dataset

**Figure 1:**
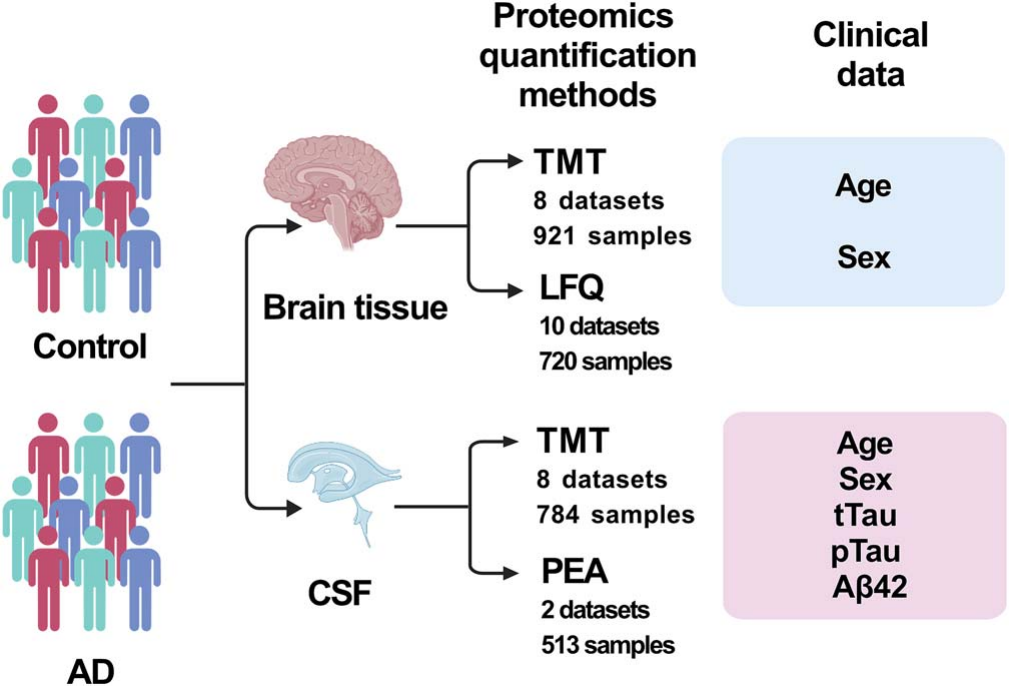
Dataset overview. The sample counts in the figure represent the total number of samples for each experimental technology and each tissue. This figure was created with BioRender.com.

This study focused on two types of samples: brain tissue and CSF (Fig. 1). For brain tissue, we used data on relative protein abundance quantified by tandem mass tag-based mass spectrometry (TMT) [10] and label-free quantitation (LFQ) [11]; datasets for these quantification methods were analyzed separately. Clinical information included sex, age, and disease state (control, AD). Samples associated with co-morbid AD and Parkinson’s disease (PD) were assigned to the AD state. Data for CSF originated from two proteomics platforms: TMT and Olink proximity extension assay (PEA) [12]; these datasets were analyzed separately. Clinical information included sex, age, disease state (control, AD), elevated total tau (tTau), 181-phosphorylated tau (pTau), and low amyloid-β42 (Aβ42). The latter three parameters are known protein-based biomarkers in AD [13]. In all cases, the assignments of disease states to samples (control and AD) were obtained from previously published research. The detailed information about each dataset is shown in Supplementary Table 1.

### Brain tissue samples

We analyzed a total of five TMT datasets for brain tissue obtained from the AD Knowledge Portal database (referred to by the AD Knowledge Portal as Banner, MSBB, Rosmap round1, Rosmap round2, and Rosmap_CognitiveResilience) and three additional TMT datasets from two published studies (Emory BA9 [14], Emory BA24 [14], and Higginbotham [15]). These datasets contain a total of 921 samples, with 3870 individual proteins covered by all of them. We also used eight LFQ datasets from the AD Knowledge Portal database (ACT, Banner, BLSA, Emory_ADRC, MSBB, MayoRNAseq, UPenn, UPennPilot) and from two additional studies (Aging [16] and Umoh [17]). There are 720 samples in these datasets, with 1311 individual proteins covered by all of them. Detailed information on each dataset (number of samples and proteins as well as clinical information) is provided in Supplementary Table 1. The number of overlapping proteins captured in different TMT and LFQ datasets is shown in Supplementary Fig. 1 and Supplementary Fig. 2.

### Cerebrospinal fluid samples

We analyzed the total of eight TMT datasets for CSF from the Synapse database (referred to by the Synapse as syn44132374 [18], syn20821165-cohort 1 [15], syn20821165-cohort 4 [15], syn20821165-discovery [15], syn20933797-cohort 1 [16], syn20933797-cohort 2 [16], syn30549757-neat [19], and syn30549757-deplete [19]). These datasets contain a total of 784 samples, with 247 individual proteins covered by all of them.

We used two PEA datasets from the Synapse database (syn52282088 [20] and syn30549757 [19]). There are 513 samples in these datasets, with 624 individual proteins covered by all of them. The number of overlapping proteins captured in different TMT and PEA datasets is shown in Supplementary Fig. 3 and Supplementary Fig. 4.

### Data pre-processing

Proteins containing missing values in more than half of the samples were excluded from consideration. Protein abundance values were log2 transformed and missing values were imputed by MissForest [21].

### Data integration and removal of intra- and inter-cohort batch effects

In this study, we compared four batch effect correction methods—ComBat [22], BBKNN [23], DESC [24], and Scanorama [25], all as implemented in the scib package [26], to identify an optimal approach for removing intra- and inter-cohort batch effects from unscaled data. Batch correction metrics (Graph Connectivity, Average silhouette width, and Principal component regression score) were utilized to measure how well the batches were mixed. The metrics were scaled between 0 and 1, with larger scores representing better batch removal. Batch correction scores were calculated as the mean of the three batch correction metrics mentioned above. To assess label conservation, we utilized silhouette score [27] and Adjusted Rand Index (ARI) [28]. Silhouette score, ranging from −1 to 1, measures how well-separated and cohesive clusters are, with higher values indicating better-defined clusters. ARI, ranging from 0 to 1, evaluates clustering consistency by comparing results to ground truth, with values closer to 1 reflecting greater agreement. For silhouette score analysis, we tested resolutions between 0.3 and 1 using the cosine metric, while ARI was evaluated across a broader resolution range of 0.1 to 2.5. Clustering was performed using the Louvain method as implemented in the Scanpy package [29]. We used the scikit-learn [30] implementation of the silhouette score and ARI.

### Combining proteomics and clinical data

We integrated two modalities—proteomics and clinical data—using multi-modal data containers as implemented in the muon package [31]. In the dataset from the brain tissue, the TMT dataset included 921 samples and 3873 features (3870 proteomics and 2 clinical features), and the LFQ dataset included 720 samples and 1314 features (1311 proteomics and 2 clinical features).

In the dataset from the CSF, the TMT dataset included 784 samples and 252 features (247 proteomics and 5 clinical features). The PEA dataset included 513 samples and 629 features (624 proteomics and 5 clinical features).

### Differential protein abundance analysis

Proteins differentially abundant between AD and control (*P* < .05) were identified using limma (version 3.46.0) [32]. Linear modeling with empirical Bayes moderation was performed using the lmFit and eBayes functions with default settings. The default parameters of limma were adopted because they are widely used and well-validated in proteomic and transcriptomic studies. Given our aim to detect potentially biologically meaningful candidate markers, we used an unadjusted *P* < .05 significance threshold to retain a broad candidate pool for downstream machine learning analysis. Differential abundance was visualized as volcano plots generated with the ggplot2 R package.

### Feature selection

To determine the optimal number of features, including clinical and proteomics data, we employed SelectFromModel as implemented in scikit-learn. Our selection criteria involved choosing features with importance scores exceeding the mean importance of all features. This procedure was performed on the training and validation datasets. Subsequently, the model exhibiting the highest balanced accuracy while maintaining a constraint of no >25 features per classification signature was selected.

### Machine learning

Combined proteomics and clinical data were used as inputs for Random Forest and Logistic Regression models to predict two disease groups: control and AD. For training and validation, we implemented a nested 5 × 5-fold cross-validation to tune hyperparameters while evaluating and comparing machine learning models. For Random Forest, hyperparameters tuned included number of trees (20–2000), tree depth (1–15, None), minimum samples to split (2, 5, 10), minimum samples per leaf (1, 2, 4), and bootstrap (True/False). For Logistic Regression the penalty (L1 or L2) was tuned. Hyperparameters were optimized using 5-fold inner cross-validation via RandomizedSearchCV (from scikit-learn), and the best model was refit on the full training set. Balanced accuracy was the optimization criterion. The class weight method [33] was employed to handle class imbalance.

The machine learning pipeline was designed to prevent data leakage. All pre-processing steps, including batch effect correction and feature selection, were carried out strictly within the training and validation folds. The test dataset was kept entirely independent and used for final evaluation, ensuring robust and generalizable results.

The classification performance for each disease group was measured using precision, recall, F1 score, balanced accuracy, area under the curve (AUC) of the receiver operating characteristic (ROC) curve, and the area under a precision-recall curve (AUC-PR). Finally, we used the explainable AI framework SHapley Additive exPlanations (SHAP) to reveal the impact of each feature on prediction scores [34].

## Results

### Overview of the study

**Figure 2:**
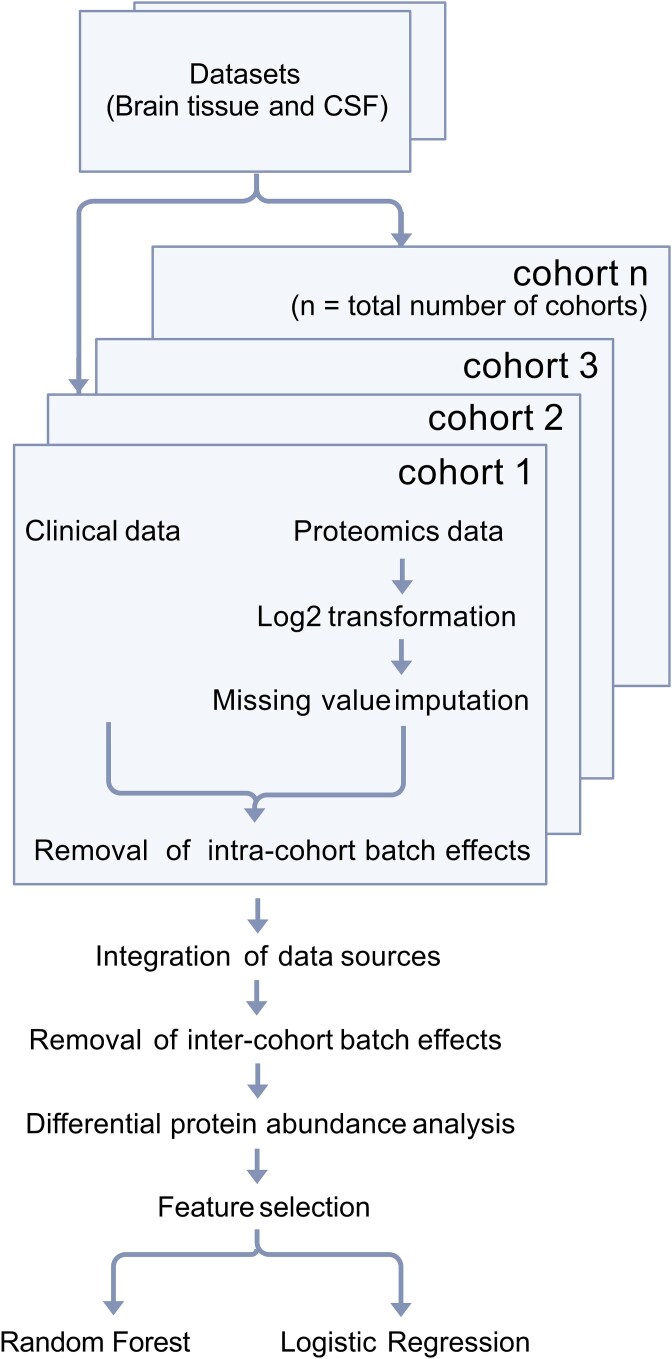
Overview of the analysis pipeline, from cohort-level preprocessing and batch effect correction to data integration, feature selection, and machine learning-based classification using random Forest and logistic regression.

This study focused on samples from two sources—brain tissue and CSF (Fig. 2). Our computational pipeline incorporates data pre-processing and batch effect correction, followed by differential protein abundance analysis, feature selection, and disease state prediction based on Random Forest and Logistic Regression. The pre-processing step involves missing value imputation in the proteomics data and the removal of intra-cohort batch effects. After combining different data sources, inter-cohort batch effects were also removed. Significantly differentially abundant proteins were selected as features for the prediction model construction. Finally, we identified the subsets of important predictive protein biomarkers to classify samples into two groups—AD and control.

### Data pre-processing

We integrated data from multiple cohorts and removed intra- and inter-cohort batch effects. To accommodate some of the batch effect correction methods that cannot process missing values, we restricted the analysis to proteins with missing values in <50% of samples within each cohort and the proteins present across all samples of all cohorts for imputation. Protein abundance values were log2 transformed. The MSBB, syn44132374, and syn52282088 datasets did not require missing value imputation, while proteomics data from other cohorts underwent imputation using MissForest. The original data for MSBB and syn44132374 had already undergone imputation, whereas for syn52282088, the reference did not indicate whether imputation was performed. Supplementary Table 1 provides details on the number of missing proteomic values across cohorts.

**Figure 3:**
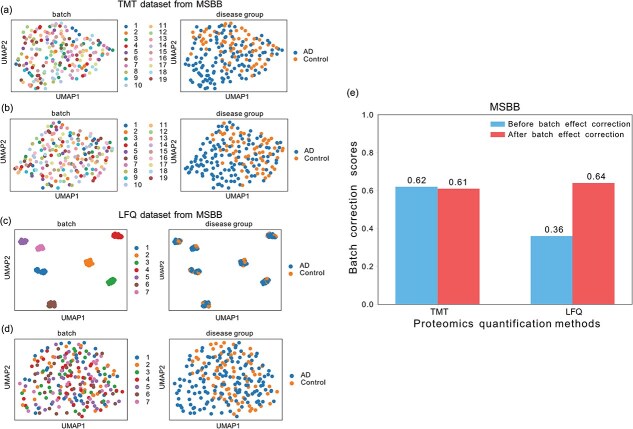
Intra-cohort batch effect correction shown for the MSBB brain tissue cohort with ComBat. (a–d) UMAP visualizations of the TMT dataset before (a) and after (b) batch effect correction, and of the LFQ dataset before (c) and after (d) batch effect correction. (e) Bar plot showing batch correction scores before and after ComBat adjustment. Numbers in UMAP plots indicate the batch ID within each dataset (see Supplementary Table 1).

Some studies in Synapse have already used TAMPOR [35] to correct for batch effects. However, certain datasets downloaded from Synapse still retain batch effects. For instance, while the TMT dataset from the MSBB cohort has undergone batch effect correction, the LFQ dataset from the same cohort has not (Fig. 3a and c). In this study, we applied ComBat for intra-cohort batch effect correction, as it is well-suited for small sample sizes and can remove batch effects across multiple groups [36]. Regardless of whether the dataset has already undergone batch effect correction, ComBat was applied to ensure homogeneous removal of batch effects. Intra-cohort batch effect processing was not conducted for cohorts lacking batch information, such as ACT, BLSA, Emory_ADRC, UPennPilot, and Umoh from the TMT dataset of brain tissue, as well as syn52282088 and syn30549757 from the PEA dataset of CSF (see Supplementary Table 1).

After applying ComBat to remove batch effects (see examples in Fig. 3b and d), the batch correction score for the LFQ dataset from the MSBB cohort was significantly improved from 0.36 to 0.64 (Fig. 3e). Five cohorts showed significant improvements in batch correction scores: Banner, Rosmap_round 2, and Higginbotham from the TMT dataset of brain tissue, as well as Banner and MSBB from the LFQ dataset of brain tissue. Supplementary Figs 5, 6, and  7 present bar plots of the batch correction scores across TMT and LFQ datasets derived from the brain tissue and CSF.

### Proteomics data integration

To address the inter-cohort batch effect impacting differential protein abundance analysis and the machine learning process, we evaluated four different batch effect correction methods (Scanorama, BBKNN, DESC, and ComBat).

### Brain tissue

**Figure 4:**
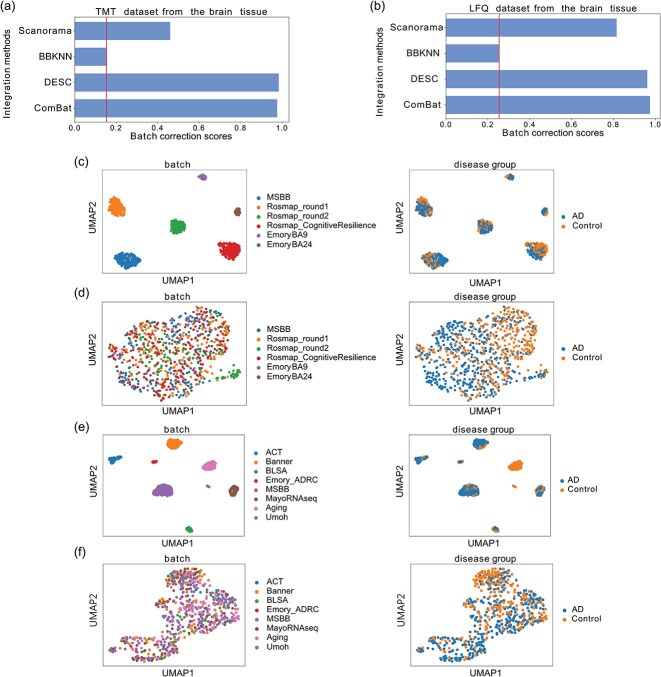
Inter-cohort batch effect correction in the TMT and LFQ datasets. (a and b) Batch correction scores for different batch effect correction methods in the TMT (a) and LFQ (b) datasets; solid lines indicate scores before correction. (c–f) UMAP visualizations of the TMT dataset before (c) and after (d) correction with DESC, as well as the LFQ dataset before (e) and after (f) correction with DESC.

In the TMT dataset, the batch correction scores were 0.9767 for ComBat, 0.9844 for DESC, 0.1536 for BBKNN, and 0.4601 for Scanorama, while the score before inter-cohort batch effect removal was 0.1536 (Fig. 4a). In the LFQ dataset, the batch correction scores were 0.9745 for ComBat, 0.9614 for DESC, 0.2542 for BBKNN, and 0.8152 for Scanorama, while the score before inter-cohort batch effect removal was 0.2542 (Fig. 4b). Figure 4c and e demonstrate the TMT and LFQ datasets before batch effect correction. DESC effectively removes the batch effect (left side of Fig. 4d and f) and separates the groups based on disease labels (right side of Fig. 4d and f).

In this study, we selected DESC over ComBat for batch effect correction across cohorts due to its superior ability to mitigate batch effects while preserving label conservation, an important metric for assessing correction efficacy. An ideal correction method should remove technical differences between cohorts while preserving distinguishable biological characteristics. To evaluate this, we utilized UMAP as a dimensionality reduction technique for visualization. By projecting corrected data into a lower-dimensional space, we assessed whether samples clustered according to their labels (e.g. disease groups) rather than exhibiting excessive overlap, which could indicate a loss of biological signal.

We illustrate this approach using the LFQ dataset (Supplementary Fig. 8), where DESC and ComBat outcomes are compared using UMAP projections. The left panels, colored by cohort, confirm that both methods effectively reduce batch effects, while the right panels, colored by disease group, reveal DESC’s advantage in maintaining well-separated disease clusters compared to ComBat. To further quantify clustering quality, we employed silhouette score and ARI, as presented in Supplementary Fig. 9 The left panel depicts the relationship between batch correction scores and ARI, while the right panel shows its correction with silhouette scores. For the TMT brain dataset, DESC achieved a silhouette score of 0.20 and an ARI of 0.52, outperforming ComBat’s 0.19 and 0.50, respectively. Similarly, in the LFQ brain dataset, DESC yielded a silhouette score of 0.36 and an ARI of 0.47, compared to ComBat’s 0.17 and 0.40. These metrics underscore the trade-offs between batch correction and clustering performance, with DESC outperforming ComBat in both silhouette score and ARI for the TMT and LFQ datasets. These findings reinforce our preference for DESC, which consistently outperforms ComBat in preserving biological signals while minimizing batch effects across TMT and LFQ datasets.

### Cerebrospinal fluid

**Figure 5:**
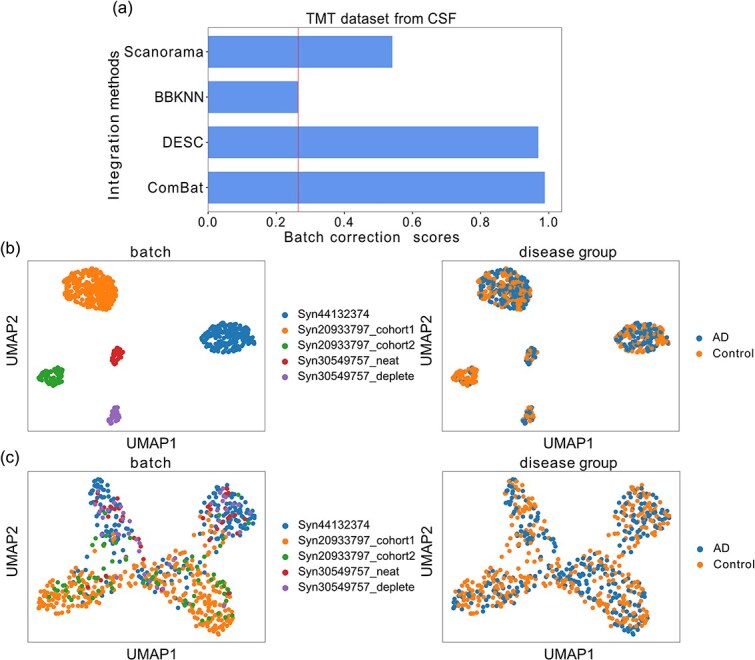
Inter-cohort batch effect correction in the CSF TMT dataset. (a) Batch correction scores for different batch effect correction methods; the solid line indicates the score before correction. (b and c) UMAP visualizations of the dataset before (b) and after (c) batch effect correction with DESC.

In the TMT dataset, the batch correction scores were 0.9881 for ComBat, 0.9691 for DESC, 0.2646 for BBKNN, and 0.5406 for Scanorama, while the score before inter-cohort batch effect removal was 0.2646 (Fig. 5a). Figure 5b demonstrates the TMT datasets before batch effect correction. DESC effectively removes the batch effect (left side of Fig. 5c) and separates the groups based on disease labels (right side of Fig. 5c).

We quantified the clustering quality of CSF datasets, consistent with our approach for brain tissue, using silhouette score and ARI, with results presented in Supplementary Fig. 9 For the TMT CSF dataset, DESC achieved a silhouette score of 0.53 and an ARI of 0.30, compared to 0.30 and 0.34 for ComBat, respectively. Given that the ARI difference between DESC and ComBat was not significant, while DESC achieved a higher silhouette score reflecting markedly better cluster separation, we selected DESC for subsequent analyses.

### Differential protein abundance

**Figure 6:**
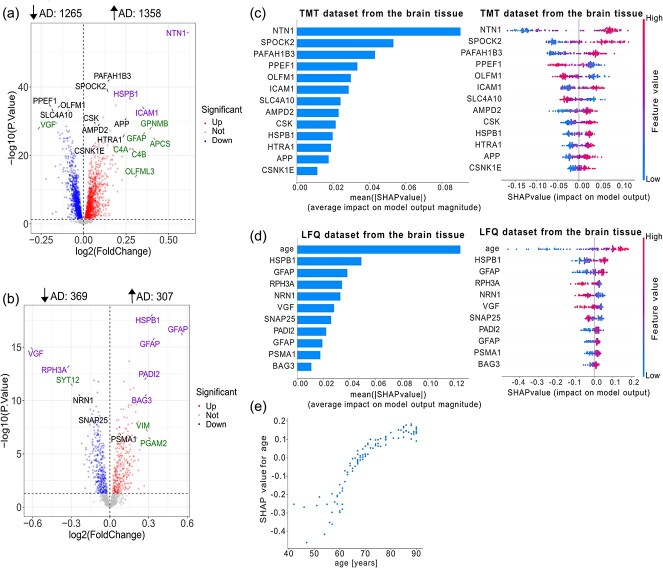
Feature selection and interpretation of Random Forest models for Alzheimer’s disease classification using brain tissue datasets. (a and b) Volcano plots for the TMT (a) and LFQ (b) datasets. Proteins selected as features are labeled and highlighted; colors indicate ML only (TMT: PPEF1, SLC4A10, OLFM1, SPOCK2, PAFAH1B3, CSK, APP, AMPD2, HTRA1, CSNK1E; LFQ: NRN1, SNAP25, PSMA1), log₂FC top 10 only (TMT: GPNMB, GFAP, APCS, C4A, C4B, OLFML3; LFQ: SYT12, VIM, PGAM2), and both ML and log₂FC top 10 (TMT: NTN1, HSPB1, ICAM1; LFQ: VGF, RPH3A, HSPB1, GFAP, GFAP, PADI2, BAG3) (see Results for detailed definitions). Numbers above each panel indicate significantly upregulated and downregulated proteins (*P*-value <.05). (c and d) Feature importance in the TMT (c) and LFQ (d) datasets is shown as summary bar plots (left) and beeswarm plots (right), with features ranked by mean SHAP values. (e) SHAP dependence plot for the LFQ dataset showing age (x-axis) versus SHAP values (y-axis), indicating that younger age lowers and older age raises the predicted risk of AD. Studies included in the TMT dataset are MSBB, Rosmap_round1, Rosmap_round2, Rosmap_CognitiveResilience, Emory BA9, and Emory BA24. Studies included in the LFQ dataset are ACT, Banner, BLSA, Emory_ADRC, MSBB, MayoRNAseq, Aging, and Umoh.

**Figure 7:**
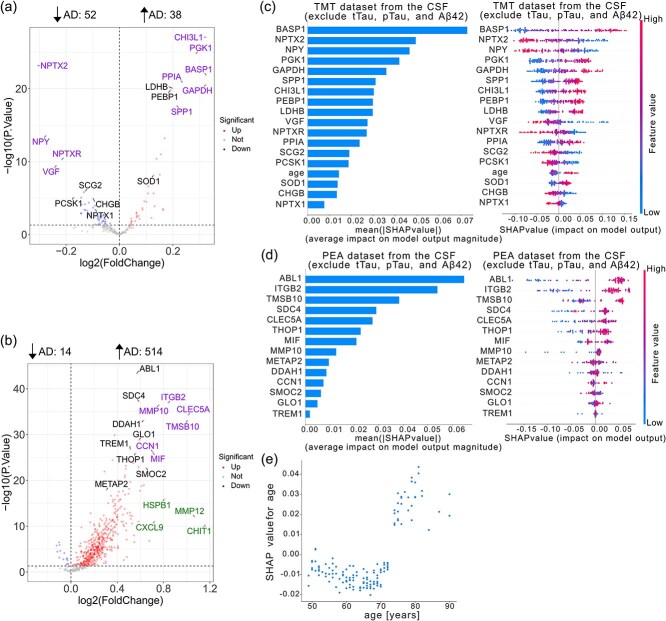
Feature selection and interpretation of Random Forest models for Alzheimer’s disease classification using CSF datasets. (a and b) Volcano plots for the TMT (a) and PEA (b) datasets. Proteins selected as features are labeled and highlighted; colors indicate ML only (TMT: SCG2, PCSK1, CHGB, NPTX1, LDHB, PEBP1, SOD1; LFQ: ABL1, SDC4, DDAH1, GLO1, TREM1, THOP1, SMOC2, METAP2) , log₂FC top 10 only (LFQ: HSPB1, CXCL9, MMP12, CHIT1), and both ML and log₂FC top 10 (TMT: NPTX2, NPY, NPTXR, VGF, CHI3L1, PGK1, BASP1, PPIA, GAPDH, SPP1; LFQ: ITGB2, MMP10, CLEC5A, TMSB10, CCN1, MIF) (see Results for detailed definitions). Numbers above each panel indicate significantly upregulated and downregulated proteins (*P*-value <.05). (c and d) Feature importance in the TMT (c) and PEA (d) datasets is shown as summary bar plots (left) and beeswarm plots (right), with features ranked by mean SHAP values. (e) SHAP dependence plot for the TMT dataset showing age (x-axis) versus SHAP values (y-axis), indicating that younger age lowers and older age raises the predicted risk of AD. Studies included in the TMT dataset are syn44132374, syn20933797 cohort 1, syn20933797 cohort 2, syn30549757 neat, and syn30549757 deplete. The study included in the PEA dataset is syn52282088.

To identify key proteins involved in AD, we focused on significantly (P-value <0.05) differentially abundant proteins between control and AD samples and used them as features for subsequent machine learning classification. In the brain tissue, there were 2623 differentially abundant proteins (down-regulated = 1265 and up-regulated = 1358) in the TMT dataset (Fig. 6a), while there were 676 differentially abundant proteins (down-regulated = 369 and up-regulated = 307) in the LFQ dataset (Fig. 6b). In the CSF samples, there were 90 differentially abundant proteins (down-regulated = 52 and up-regulated = 38) from the TMT dataset with the criteria of P.Value <0.05 (Fig. 7a). In comparison, 528 differentially abundant proteins (down-regulated = 14 and up-regulated = 514) were from the PEA dataset (Fig. 7b). The lists of differentially abundant proteins can be found in the Supplementary Tables 2, 3, 4, and  5.

### Machine learning

To develop robust control/AD classifiers, we first addressed cross-cohort reproducibility. The 28 cohorts in this study span proteomic platforms with substantial technical differences (TMT, LFQ, PEA). To maintain technical consistency, cohorts were merged only within each platform. Models were trained using nested cross-validation within the training sets, while independent cohorts not used during training served as test sets to evaluate model performance and the consistency of the biomarker panels.

Differentially abundant proteins were used as features to develop a control/AD classifier using Random Forest and Logistic Regression models. We employed separate datasets for training, validation, and testing. For the TMT brain tissue dataset, training and validation data included MSBB, Rosmap_round1, Rosmap_round2, Rosmap_CognitiveResilience, Emory BA9, and Emory BA24, while the testing data included Banner and Higginbotham. In the LFQ brain tissue dataset, training and validation data consist of ACT, Banner, BLSA, Emory_ADRC, MSBB, MayoRNAseq, Aging, and Umoh, while the testing data includes UPenn and UPennPilot. For the TMT CSF dataset, training and validation data included syn44132374, syn20933797_cohort 1, syn20933797_cohort 2, syn30549757_neat, syn30549757_deplete, while the testing data included syn20821165_cohort 1, syn20821165_cohort 4, and syn20821165_discovery. In the PEA CSF dataset, syn52282088 was used for training and validation data, and syn30549757 for testing.

Classification performance for each disease group was evaluated using precision, recall, F1 score, balanced accuracy, AUC-ROC, and AUC-PR. Additionally, SHAP values were used to assess the impact of each feature on prediction scores, with results visualized through summary bars and beeswarm plots.

### Brain tissue

**Figure 8:**
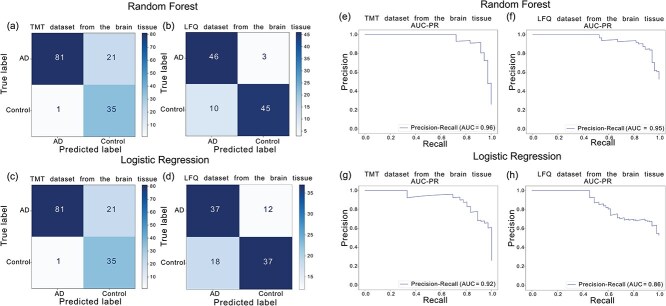
Performance of Random Forest and Logistic Regression models in classifying Alzheimer’s disease and healthy controls based on brain tissue hold-out sets. (a–d) Confusion matrices for Random Forest on TMT (a) and LFQ (b) datasets, and for Logistic Regression on TMT (c) and LFQ (d) datasets. (e–h) Precision-recall curves for random Forest on TMT (e) and LFQ (f), and for Logistic Regression on TMT (g) and LFQ (h) datasets.

In this study, we employed Random Forest and Logistic Regression models to classify AD and control samples in the TMT and LFQ datasets and subsequently evaluated their classification performance. Features were selected based on their model-derived importance weights using the SelectFromModel method. The Random Forest model achieved an AUC-PR of 0.96 with 13 features on the TMT dataset and 0.95 with 11 features on the LFQ dataset (Table 1). Logistic Regression reached an AUC-PR of 0.92 (20 features) on TMT and 0.86 (24 features) on LFQ. Figure 8 presents the confusion matrices and AUC-PR curves for the best-performing classifiers on the hold-out set. All models consistently achieved performance scores near or above 0.9.

**Table 1:** Classification performance of Random Forest and Logistic Regression models in distinguishing AD from control samples, based on brain tissue proteomics data from two platforms: TMT and LFQ.

**TMT dataset from the brain tissue** **(*n* = 138)**					
	**features = 13^a^**				
**Classifier**	**Balanced accuracy** **(95% CI)**	**AUC-ROC** **(95% CI)**	**AUC-PR** **(95% CI)**	**Sensitivity** **(95% CI)**	**Specificity** **(95% CI)**
Random Forest (cross-validation)	0.87 (0.85-0.90)	0.94 (0.91-0.96)	0.93 (0.90-0.95)	0.90 (0.87-0.94)	0.84 (0.78-0.89)
Random Forest (test)	0.88 (0.83-0.92)	0.98 (0.95-0.99)	0.96 (0.91-0.99)	0.97 (0.90-1)	0.79 (0.70-0.86)
	**features = 20^b^**				
Logistic Regression (cross-validation)	0.83 (0.75-0.86)	0.91 (0.86-0.93)	0.89 (0.86-0.92)	0.84 (0.77-0.88)	0.81 (0.73-0.87)
Logistic Regression (test)	0.88 (0.83-0.92)	0.97 (0.93-0.98)	0.92 (0.83-0.97)	0.97 (0.91-1)	0.79 (0.71-0.86)
**LFQ dataset from the brain tissue** **(*n* = 104)**					
	**features = 11^c^**				
**Classifier**	**Balanced accuracy** **(95% CI)**	**AUC-ROC** **(95% CI)**	**AUC-PR** **(95% CI)**	**Sensitivity** **(95% CI)**	**Specificity** **(95% CI)**
Random Forest (cross-validation)	0.96 (0.89-0.99)	0.98 (0.93-0.99)	0.98 (0.93-0.99)	0.95 (0.86-0.99)	0.97 (0.92-1)
Random Forest (test)	0.88 (0.81-0.93)	0.93 (0.88-0.97)	0.95 (0.90-0.98)	0.81 (0.70-0.91)	0.93 (0.86-1)
	features = 24^d^				
Logistic Regression (cross-validation)	0.72 (0.70-0.75)	0.80 (0.73-0.84)	0.76 (0.62-0.84)	0.71 (0.68-0.76)	0.74 (0.65-0.80)
Logistic Regression (test)	0.71 (0.63-0.79)	0.82 (0.74-0.89)	0.86 (0.77-0.92)	0.67 (0.54-0.78)	0.75 (0.63-0.87)

^a^The 13 features selected for the TMT dataset include AMPD2, APP, CSK, CSNK1E, HSPB1, HTRA1, ICAM1, NTN1, OLFM1, PAFAH1B3, PPEF1, SLC4A10, and SPOCK2.

^b^The 20 features selected for the TMT dataset include ABCB1, ACOT8, ADGRB1, ADGRB3, APOE, ATAT1, FIS1, HLA-DRA, MPDZ, NCBP2, NLGN3, NTN1, PABPC1L2A/PABPC1L2B, PDPR, PSMB5, RAB27B, RPL35, RWDD1, STXBP1, and TAB3.

^c^For the LFQ dataset, the 11 selected features are age, VGF, BAG3, HSPB1, GFAP (P14136), PSMA1, SNAP25, NRN1, RPH3A, PADI2, and GFAP (K7EKD1).

^d^For the LFQ dataset, the 24 selected features are IPO5, MGST3, VPS26A, APOE, RALB, TALDO1, RPS27, LRPPRC, CRIP2, EIF5, YWHAH, LRP1, RAB35, PPP1R7, GDAP1, CUL5, KCTD12, BDH2, ACAD9, PLCB1, LANCL2, PSME2, ATP6V1D, and SYT1.

To further explore the key features, we visualized their distribution using a volcano plot of differential abundance, with the Random Forest model serving as a representative example (Fig. 6a and b). For differential abundance analysis the top 10 proteins ranked by log₂FC with *P* < .05, were categorized into three groups: (i) proteins identified exclusively by the machine learning that were not among the log₂FC top 10, shown in black; (ii) proteins within the top 10 according to log₂FC, but absent from the machine learning results, shown in green; and (iii) proteins within the log₂FC top 10 and also identified by the machine learning, shown in violet. Supplementary Table 6 provides detailed information on the differential protein abundance of these critical features, as predicted by the Random Forest model, in both the TMT and LFQ datasets.

Furthermore, Fig. 6c and d illustrate the contribution of individual features to the classification of each disease group in both the TMT and LFQ datasets, with Random Forest being used as the model of choice. Notably, age is a significant predictor in the Random Forest model for the LFQ dataset, but not for the TMT dataset. To further investigate this, we utilized the SHAP dependence plot to examine the influence of age on the predictions. As shown in Fig. 6e, older age is associated with higher prediction accuracy for AD in the LFQ dataset.

### Cerebrospinal fluid

tTau, pTau, and Aβ42 are well-established biomarkers for AD [5]. To evaluate the added value of other features, we assessed the performance of our Random Forest and Logistic Regression models under two scenarios: one excluding tTau, pTau, and Aβ42, and another using only these biomarkers as input features. Furthermore, our subsequent analyses focus exclusively on models excluding tTau, pTau, and Aβ42, aiming to identify potential novel biomarkers beyond the established ones.

The Random Forest model achieved an AUC-PR of 0.98 with 18 features on the TMT dataset and 0.88 with 14 features on the PEA dataset with tTau, pTau, and Aβ42 excluded (Table 2). Logistic Regression reached an AUC-PR of 0.95 (24 features) on TMT and 1.00 (16 features) on PEA under the same conditions.

**Table 2:** Classification performance of Random Forest and Logistic Regression models in distinguishing AD from control samples, based on CSF proteomics data from two platforms: TMT and PEA.

**TMT dataset from the CSF** **(*n* = 139)**															
	**Without tTau, pTau, Aβ42** (features = 18^*^)							**Only tTau, pTau, Aβ42** **(feature = tTau)**							
**Classifier**	**Balanced Accuracy** **(95% CI)**		**AUC-ROC** **(95% CI)**	**AUC-PR** **(95% CI)**	**Sensitivity** **(95% CI)**	**Specificity** **(95% CI)**		**Balanced Accuracy** **(95% CI)**		**AUC-ROC** **(95% CI)**	**AUC-PR** **(95% CI)**	**Sensitivity** **(95% CI)**		**Specificity** **(95% CI)**	
Random Forest (cross-validation)	0.96 (0.92-0.98)		0.99 (0.96-0.99)	0.99 (0.97-0.99)	0.95 (0.90-0.99)	0.97 (0.93-1)		0.81 (0.76-0.85)		0.89 (0.83-0.92)	0.91 (0.86-0.94)	0.75 (0.73-0.80)		0.87 (0.79-0.91)	
Random Forest (test)	0.88 (0.82-0.92)		0.94 (0.90-0.97)	0.98 (0.95-0.99)	0.81 (0.73-0.87)	0.94 (0.86-1)		0.95 (0.91-0.97)		0.98 (0.95-0.99)	0.99 (0.98-0.99)	0.9 (0.83-0.95)		1 (1-1)	
	(features = 24^**^)							**(features = tTau, pTau, Aβ42)**							
Logistic Regression (cross-validation)	0.95 (0.88-0.94)		0.97 (0.95-0.98)	0.97 (0.96-0.98)	0.92 (0.83-0.96)	0.92 (0.90-0.96)		0.95 (0.94-0.96)		0.98 (0.97-0.98)	0.98 (0.98-0.99)	0.94 (0.91-0.98)		0.96 (0.94-0.99)	
Logistic Regression (test)	0.80 (0.72-0.87)		0.90 (0.83-0.95)	0.95 (0.90-0.98)	0.78 (0.70-0.85)	0.82 (0.68-0.93)		0.92 (0.87-0.96)		0.99 (0.97-0.99)	1 (0.98-1)	0.87 (0.80-0.93)		0.97 (0.91-1)	
**PEA dataset from the CSF** **(*n* = 88)**															
	**Without tTau, pTau, Aβ42** (features = 14^***^)					**Only tTau, pTau, Aβ42** **(feature = Aβ42)**					**With tTau, pTau, Aβ42** **(features = Aβ42)**				
**Classifier**	**Balanced Accuracy** **(95% CI)**	**AUC-ROC** **(95% CI)**	**AUC-PR** **(95% CI)**	**Sensitivity** **(95% CI)**	**Specificity** **(95% CI)**	**Balanced Accuracy** **(95% CI)**	**AUC-ROC** **(95% CI)**	**AUC-PR** **(95% CI)**	**Sensitivity** **(95% CI)**	**Specificity** **(95% CI)**	**Balanced Accuracy** **(95% CI)**	**AUC-ROC** **(95% CI)**	**AUC-PR** **(95% CI)**	**Sensitivity** **(95% CI)**	**Specificity** **(95% CI)**
Random Forest (cross-validation)	0.96 (0.88-0.99)	0.98 (0.93-0.99)	0.98 (0.93-0.99)	0.97 (0.90-1)	0.95 (0.85-1)	0.99 (0.98-0.99)	1 (1-1)	1 (1-1)	0.99 (0.97-1)	0.98 (0.97-1)	0.99 (0.98-0.99)	1 (1-1)	1 (1-1)	0.99 (0.97-1)	0.98 (0.97-1)
Random Forest (test)	0.62 (0.57-0.67)	0.62 (0.48-0.72)	0.88 (0.80-0.94)	0.24 (0.14-0.34)	1 (1-1)	0.50 (0.5-0.5)	0.50 (0.5-0.5)	0.90 (0.85-0.94)	0 (0-0)	1 (1-1)	0.50 (0.5-0.5)	0.50 (0.5-0.5)	0.90 (0.85-0.94)	0 (0-0)	1 (1-1)
	(features = 16^****^)					**(features = tTau, pTau)**					**(features = age, pTau, VIM)**				
Logistic Regression (cross-validation)	0.97 (0.9-1)	0.99 (0.97-1)	0.99 (0.97-1)	0.97 (0.87-1)	0.98 (0.94-1)	0.99 (0.98-1)	0.99 (0.98-1)	0.99 (0.97-1)	0.99 (0.97-1)	0.99 (0.97-1)	0.97 (0.93-0.99)	0.99 (0.98-1)	0.99 (0.98-1)	0.98 (0.94-1)	0.96 (0.92-0.99)
Logistic Regression (test)	0.69 (0.63-0.75)	0.99 (0.97-1)	0.99 (0.99-1)	0.38 (0.26-0.51)	1 (1-1)	0.50 (0.5-0.5)	0.89 (0.79-0.96)	0.97 (0.92-0.99)	1 (1-1)	0 (0-0)	0.79 (0.67-0.91)	0.87 (0.77-0.95)	0.96 (0.92-0.99)	0.92 (0.85-0.98)	0.66 (0.43-0.89)

^a^The 18 features selected for the TMT dataset include age, CHGB, SPP1, SCG2, VGF, NPTXR, CHI3L1, GAPDH, LDHB, PGK1, NPTX1, PEBP1, BASP1, SOD1, NPTX2, PCSK1, PPIA, and NPY.

^b^The 24 features selected for the TMT dataset include TF, SPP1, FGB, SCG2, ITIH1, APOA4, VGF, C2, B4GAT1, CTSD, MCAM, NPTXR, CHI3L1, ENO2, GAPDH, IGFBP2, TGOLN2, LUM, CD14, BASP1, RNASE4, PCSK1, FRZB, and NPY.

^c^For the PEA dataset, the 14 selected features are SMOC2, GLO1, MMP10, ITGB2, CCN1, METAP2, ABL1, TREM1, THOP1, CLEC5A, SDC4, DDAH1, TMSB10, and MIF.

^d^For the PEA dataset, the 16 features selected are OLR1, MMP10, ITGB2, DLK1, SPON1, ABL1, TREM1, THOP1, DDC, SDC4, ROR1, DDAH1, TMSB10, ESAM, ENTPD6, and VEGFA.

**Figure 9:**
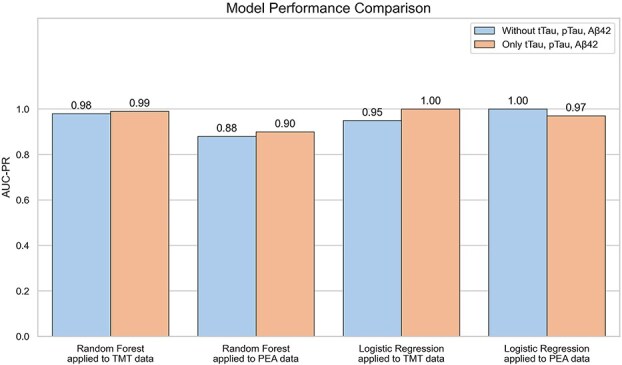
Comparison of model performance based on AUC-PR curves using data generated from different proteomic quantification methods and machine learning methods. The barplot illustrates the results of two classification methods—Random Forest and Logistic Regression—applied to TMT and PEA proteomics datasets. Each bar represents the performance measured on two different groups. For each pair of bars, the left bar represents “Without tTau, pTau, Aβ42”, and the right bar represents “Only tTau, pTau, Aβ42”. The y-axis shows AUC-PR curves ranging from 0 to 1.0. The x-axis labels indicate the classification method and data type.

**Figure 10:**
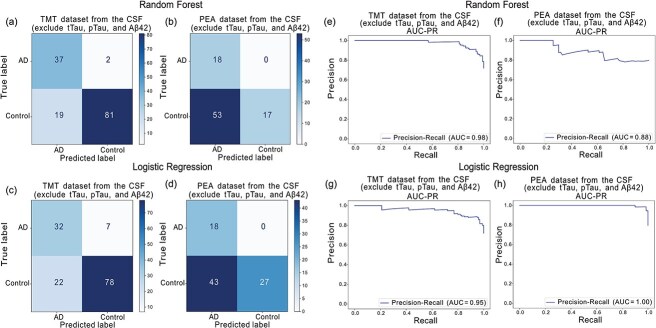
Performance of Random Forest and Logistic Regression models for classifying Alzheimer’s disease and healthy controls based on CSF hold-out set. (a–d) Confusion matrices for the Random Forest on TMT (a) and PEA (b) datasets, and for logistic regression on TMT (c) and PEA (d) datasets. (e–h) Precision-recall curves for random Forest on TMT (e) and PEA (f), and for Logistic Regression on TMT (g) and PEA (h) datasets.

Using only tTau, pTau, and Aβ42 as features, the Random Forest model achieves an AUC-PR of 0.99 with a single feature (tTau) on the TMT dataset and 0.90 with Aβ42 alone on the PEA dataset. Logistic Regression reaches an AUC-PR of 1.00 using all three biomarkers on TMT, and 0.97 with tTau and pTau on PEA. Figure 9 shows the comparison of model performance based on AUC-PR curves using data generated from different proteomic quantification methods and machine learning methods. Figure 10 shows the confusion matrices and AUC-PR curves for the best-performing models. Overall, performance consistently approaches or exceeds an AUC-PR of 0.9 across all settings.

tTau, pTau, and Aβ42 improve balanced accuracy in the TMT dataset but not in the PEA dataset. This difference may be due to the PEA test set containing only 18 AD samples and 70 controls. The small sample size and class imbalance could have contributed to unstable results. Consequently, we included these biomarkers alongside clinical and proteomics features in both Random Forest and Logistic Regression models. In the Random Forest model, balanced accuracy remained at 0.50 even after incorporating these biomarkers. In contrast, the Logistic Regression model achieved a balanced accuracy of 0.79 using age, pTau, and VIM.

To further explore key features, we visualized their distribution on a volcano plot of differential abundance using the Random Forest model as a representative example (Fig. 7a and b) and the same color code as in (Fig. 6a and b). Supplementary Table 7 provides detailed differential abundance data for these features in the TMT and PEA datasets. Feature contributions to disease group classification are shown in Fig. 7c and d, again using the Random Forest model as an example. Age is a key predictor in the Random Forest model for the TMT dataset, but not for PEA. To explore this further, we used a SHAP dependence plot to assess age’s influence on model predictions. As shown in Fig. 7e, prediction accuracy for AD increases with age in the TMT dataset.

## Discussion

In this study, a computational approach was applied to integrate publicly available proteomics datasets from AD samples. In total, 8 TMT and 10 LFQ datasets from brain tissue and 8 TMT and 2 PEA datasets from CSF were included. For brain tissue and CSF independently, classification was performed using Random Forest and Logistic Regression models.

For brain tissue, Random Forest selected 13 proteins as predictive features for the TMT and 10 for the LFQ datasets. Logistic Regression selected 20 proteins for the TMT and 24 for the LFQ datasets. For CSF, Random Forest selected 17 predicting proteins for the TMT and 14 for the PEA datasets. Logistic Regression selected 24 proteins for the TMT and 16 for the PEA datasets.

Supplementary Fig. 10a and b show the overlap between different machine learning methods and different proteomics quantification methods for brain tissue and CSF, respectively. There is almost no overlap for brain tissue, either between machine learning or between mass spectrometric technologies. Brain tissue exhibits high biological heterogeneity, comprising diverse cell types such as neurons, astrocytes, and microglia, and AD pathology varies across brain regions. Consequently, protein expression is highly region- and cell type–specific, reducing overlap across cohorts, proteomic platforms, and analytical methods. In contrast, for CSF, Random Forest and Logistic Regression show a considerable overlap of protein features. CSF reflects an integrated physiological signal of the central nervous system rather than localized brain signals, resulting in more consistent protein alterations that are easier to reproduce across cohorts and platforms [37, 38]. This suggests that proteomic analysis of CSF provides more consistent results across analytical strategies. Even on the same dataset, different machine learning models may produce non-overlapping protein panels. Random Forest, as a nonlinear tree-based model, can capture complex interactions and cumulative effects among proteins, whereas logistic regression detects only univariate linear associations with the outcome. Thus, each method may identify different proteins in high-dimensional biological datasets. Importantly, all selected features originate from the same altered proteins, ensuring that each protein shows at least some disease association; the lack of overlap reflects methodological differences rather than an absence of biological signal. Similar divergence among biomarker panels from different machine learning models has also been reported in AD proteomics research, such as the analysis by Tandon et al [39].

To systematically evaluate the biological relevance of the identified features, all protein candidates selected across platforms and methods were classified into four categories: (i) key biomarkers with a well-established role in AD, (ii) proteins with evidence supporting their involvement in AD, (iii) newly identified protein features without prior association, and (iv) known key AD biomarkers that were notably absent in the present analyses. This stratification explains overlapping and unique protein features in terms of the potential of biomarkers and biological significance.

For the brain tissue, 13 and 20 protein features were selected from the TMT data by Random Forest and Logistic Regression, while 11 and 24 protein features were selected from the LFQ data, respectively. A comparison of these different selections, either between computational analysis or between mass spectrometric methods, shows that there is very little overlap. For literature comparison, the NeuroPro database [40] compiles 38 proteomics studies from Alzheimer’s brain tissue. The NeuroPro database consists of 848 proteins with consistent alterations in AD in at least five studies. In addition, each protein in NeuroPro is scored by the number of publications reporting it as significantly altered (maximum: 38). Comparing our results to NeuroPro shows that our analyses identify the top 3 proteins with the highest score and five proteins in the top 10. In total, with our computational approach, we selected 35 proteins that were also present in the NeuroPro database (see Supplementary Table 8). It becomes clear that Random Forest identifies prominent protein biomarkers for AD, including GFAP, APP, HSPB1, VGF, and RPH3A. In contrast, 26 out of 44 proteins selected by Logistic Regression are not present at all among the 848 proteins in the NeuroPro database and are, besides APOE, not well known to be associated with AD (see Supplementary Table 9). An enrichment analysis on specifically those 26 proteins shows the most prominent and significant results for GO cellular compartment, with five GO terms related to synapse location. For those 26 proteins, only two proteins receive >10 PubMed hits when searching for the protein name + the term ‘Alzheimer’s Disease’. This is ABCB1, described to have a role in the transport of Aβ peptides out of the brain. Secondly, FIS1, which is described as upregulated in models of AD.

The most prominently described proteins in the AD brain that were not found in our analysis were CD44 and CLU, both of which have been reported in over 20 studies included in the NeuroPro database, because they were not present in our dataset. This indicates that constraints in sample sources may hinder the detection of certain features. However, our model, built using data from multiple independent cohorts, demonstrates that the selected protein panel still exhibits robust classification performance in distinguishing AD from controls, despite incomplete protein coverage.

For CSF, 18 and 24 protein features were selected from the TMT data by Random Forest and Logistic Regression, while 14 and 16 protein features were selected from the PEA data, respectively. Overall, there is no overlap between TMT and PEA features. This may not be surprising, as the methods largely differ, being a mass spectrometry-based untargeted and an antibody-based targeted proteomics method, respectively. Comparison of Random Forest and Logistic Regression shows considerable overlap in protein features for CSF, with roughly one-third of proteins being common between both computational strategies.

In a recent large-scale proteomic study, Ali *et al.* [6] analyzed CSF samples from 2286 participants across four cohorts, quantifying 7029 proteins. They identified 1164 novel AD-associated proteins and grouped them into four pseudo-trajectories enriched at different stages along the AD continuum. Oh *et al.* [41] performed a large-scale proteomic analysis of CSF from 3397 individuals and identified a novel biomarker—the ratio of the synaptic proteins YWHAG to NPTX2—which was found to increase significantly in association with cognitive decline in individuals with AD. In the current study, we identified several biomarkers using different proteomics quantification methods, some of which have been previously validated, while others have not yet been reported in the literature. For example, using the TMT dataset from CSF and applying a Random Forest model, we identified 17 proteins as important features. Among these, SSP1 [42], SCG2 [43], VGF [44], NPTXR [43], CHI3L1 [45], GAPDH [46], NPTX1 [47], NPTX2 [47], PCSK1 [48], NPY [49], CHGB [50], PEBP1 [51], and BASP1 [52] have already been validated in prior studies. However, the following proteins have not yet been published as AD-related biomarkers: LDHB, PGK1, SOD1, and PPIA. According to g:Profiler pathway enrichment analysis (adjusted *P* < .05, term size 2–200, intersection ≥2) [53], SOD1 and PPIA are involved in oxidative stress-induced intrinsic apoptotic pathways, while LDHB and PGK1 are associated with glycolysis/gluconeogenesis. These enriched pathways have been reported to be associated with AD [54–56].

Furthermore, our study employed multiple proteomics quantification methods, among which the antibody-based targeted proteomics approach, PEA, revealed noteworthy observations. Unlike untargeted mass spectrometry (MS)-based TMT analyses and LFQ for brain tissue, PEA targets a predefined set of proteins, with the proteins detected depending on the chosen assay panels. Two independent cohorts in this study utilized the Olink Target 96 panels: Vromen *et al.* applied 11 panels, analyzing 979 unique proteins [57], while Dammer *et al.* used 13 panels, covering 1160 unique proteins [19]. The panels employed in these studies showed only minor differences. Comparing our findings with Binette *et al.*, who used the Olink Explore 3072 panel (comprising eight multiplex panels), reveals a high degree of overlap in selected protein features [37]. Our Random Forest model identified 14 protein features, of which 13 were also detected by Binette *et al.*, with 10 proteins exhibiting significant differential abundance in their study as well. Notably, TREM1—a protein selected in our study from the Olink Target 96 immune response panel—was not detected in Binette *et al.*’s Olink Explore 3072 analysis. Furthermore, Binette *et al.*’s broader panel included many proteins not covered by the Target 96 panels, which naturally were not detected in our analysis.

### Methodological strengths and limitations

This study presents a systematic computational pipeline to overcome common challenges encountered in multi-source omics analyses. We systematically integrated proteomic data derived from brain tissue and CSF, encompassing multiple quantification methods including TMT, LFQ, and PEA. Through batch effect correction and data preprocessing, we substantially enhanced data consistency and comparability, thereby strengthening the reliability and robustness of our analyses. By combining differential protein abundance analysis with machine learning approaches—Random Forest and Logistic Regression—we developed predictive models to accurately distinguish AD patients from controls.

Matthijs B. de Geus *et al.* [58] analyzed CSF proteomics data from multiple platforms, performing enrichment analyses on the top differentially expressed proteins. Our study also integrates multi-platform CSF data using machine learning to identify protein panels that distinguish AD patients from controls, including both validated and novel candidates. Furthermore, compared to most previous single biospecimen studies often overlook critical clinical covariates such as age and sex [7, 59, 60], our approach integrates multiple data sources and clinical variables, significantly improving model accuracy and clinical relevance. To preserve the biological signals of age and sex, we combined clinical and proteomics data before batch correction and included age and sex as model features instead of covariates to retain meaningful biological variation. This allows the model and SHAP analysis to represent their contributions in the predictions. Our predictive models consistently achieve AUC-PR ≥ 0.9 across platforms, demonstrating excellent predictive performance. Integrating multi-source data and feature selection enhances the translational potential of the identified protein biomarkers.

The slightly lower performance of the model on the PEA CSF dataset may reflect several factors. First, platform differences: PEA targets specific proteins and has limited coverage, whereas MS measures a broader range. Second, differences in protein coverage and information content: PEA measures only a few hundred pre-selected proteins, while the integrated dataset contains 624 proteins. Although the TMT CSF dataset includes only 247 proteins, these likely include more key AD markers, whereas the PEA CSF dataset may lack critical biomarkers, reducing classification ability. Third, the PEA CSF dataset comprises only 2 cohorts, totaling 513 samples (training/validation = 425, independent test = 88), which may contribute to the slightly lower performance. Moreover, since the sensitivity and specificity of Random Forest and Logistic Regression on the PEA CSF dataset show opposite trends, future work could consider ensemble averaging or majority voting to improve stability and reduce individual model bias.

The current dataset comprehensively represents the publicly available samples in the AD Knowledge Portal and Synapse databases, and the identified biomarkers may have potential for early diagnosis or risk assessment. However, clinical translation still faces challenges, such as data reproducibility, biological interpretability, and population heterogeneity. We focused on AD prediction as a binary classification task (Control versus AD) and did not consider disease subtypes or progression stages, such as MCI to AD, because the available data are currently insufficient to develop classifiers for either disease subtypes or different stages of disease progression. Future multicenter studies will evaluate the model’s stability across diverse cohorts and platforms. Additionally, expanding sample size (including different AD subgroups and stages), integrating additional data types, and externally validating in larger cohorts will help confirm generalizability.

**Key Points**

- Combining large-scale proteomics datasets enables comprehensive discovery of biomarkers for Alzheimer’s disease (AD).
- Integrating proteomic data with clinical features enhances the ability of machine learning models to predict AD.
- Random Forest and Logistic Regression models effectively distinguished Alzheimer’s patients from healthy controls, consistently achieving high performance (AUC-PR ≥ 0.9) and identifying both known and novel biomarker candidates.

## Supplementary Material

bbag012_Supplemental_Files

## Data Availability

The code used for data analysis in this study is publicly available on GitHub at: https://github.com/wytsai8/DEEPROAD. All datasets can be downloaded from the AD Knowledge Portal (https://adknowledgeportal.synapse.org/) and Synapse (https://www.synapse.org/Synapse:syn5550382).
