# Supplementary material for: Multi-platform integration of brain and CSF proteomes reveals biomarker panels for Alzheimer’s disease
Source: Brief Bioinform. 2026 Jan 29;27(1):bbag012. doi: 10.1093/bib/bbag012 (PMC12853123; doi:10.1093/bib/bbag012)
Supplement: bbag012_Supplemental_Files [file bbag012_supplemental_files.zip › Supplementary_materials_bbag012.docx]

**Supplementary Material**


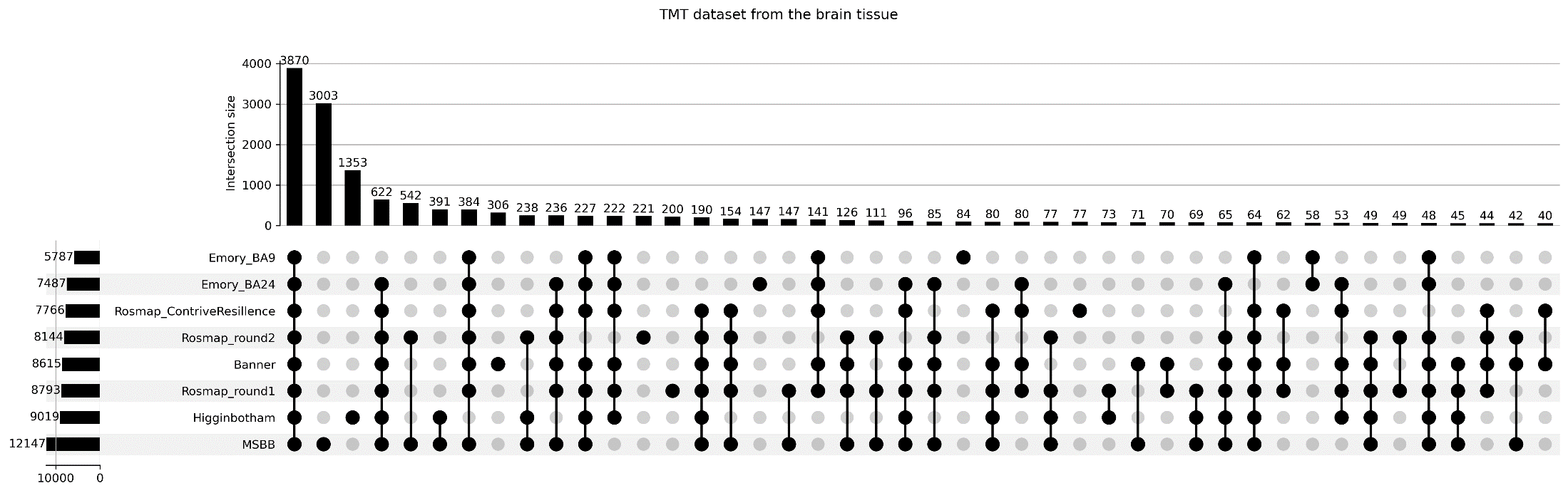


**Supplementary Figure 1.** The number of overlapping proteins captured in the different studies of the TMT dataset from the brain tissue.

**Alt text:** An UpSet plot showing the intersections of proteins across multiple TMT brain tissue studies.


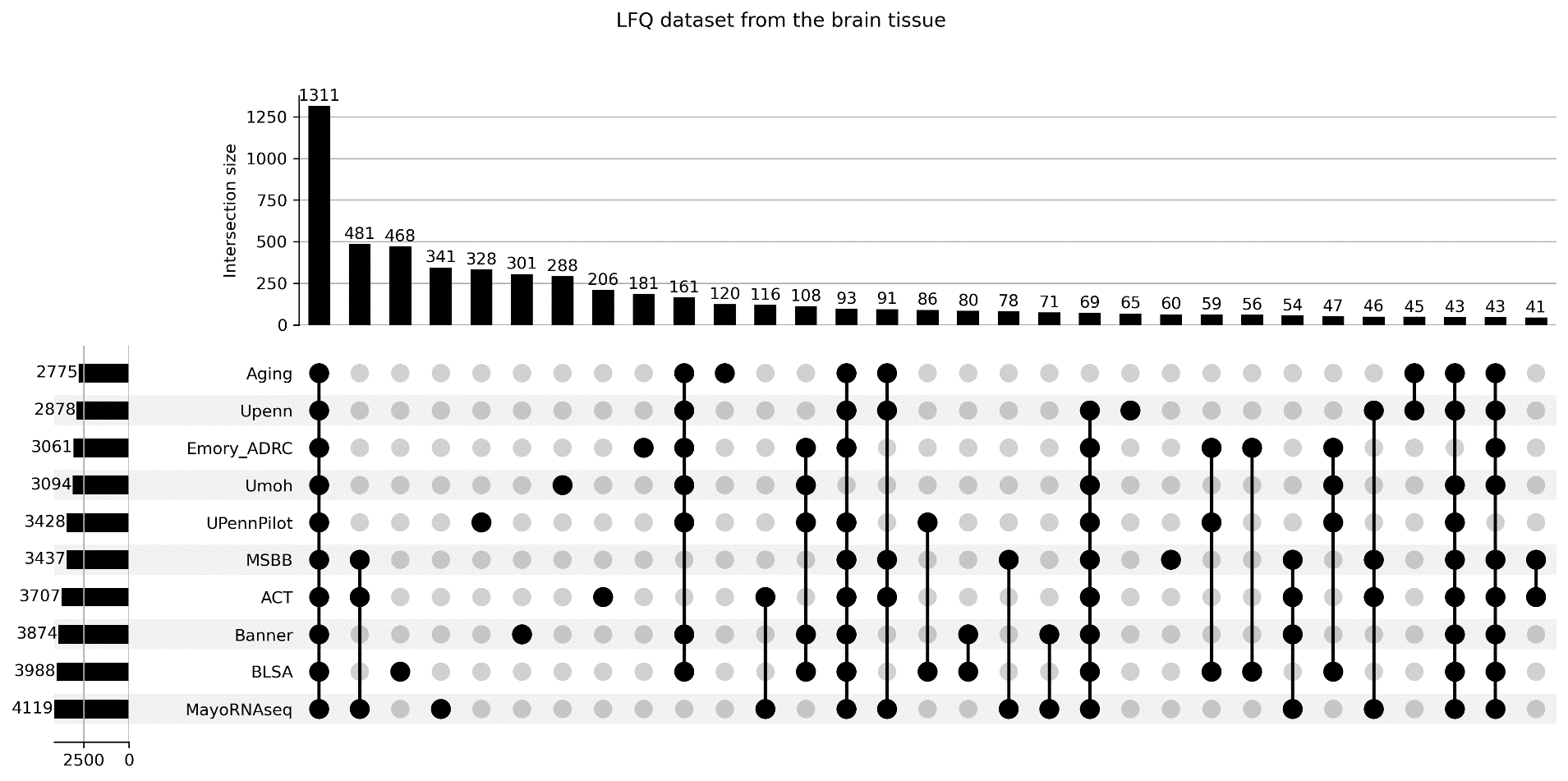


**Supplementary Figure 2.** The number of overlapping proteins captured in the different studies of the LFQ dataset from the brain tissue.

**Alt text:** An UpSet plot showing the intersections of proteins across multiple LFQ brain tissue studies.


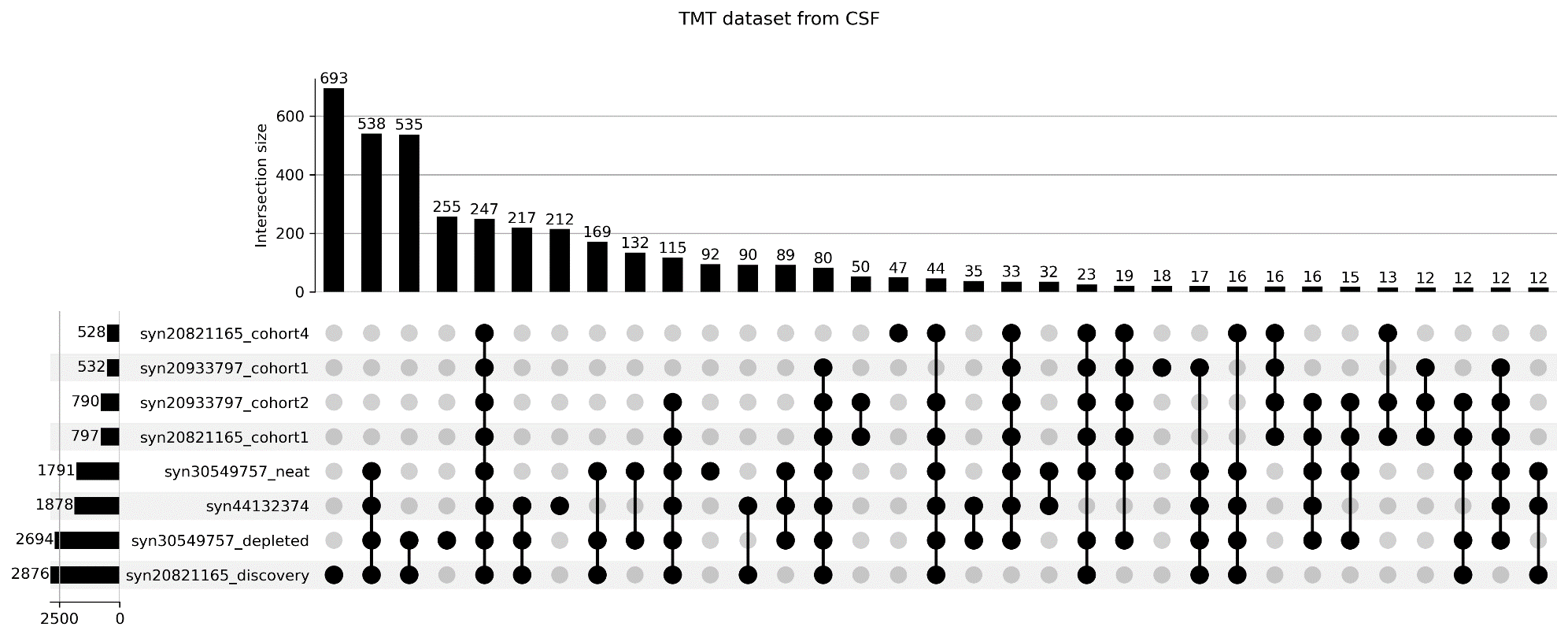


**Supplementary Figure 3.** The number of overlapping proteins captured in the different studies of the TMT dataset from the CSF.

**Alt text:** An UpSet plot showing the intersections of proteins across multiple TMT CSF studies.

**
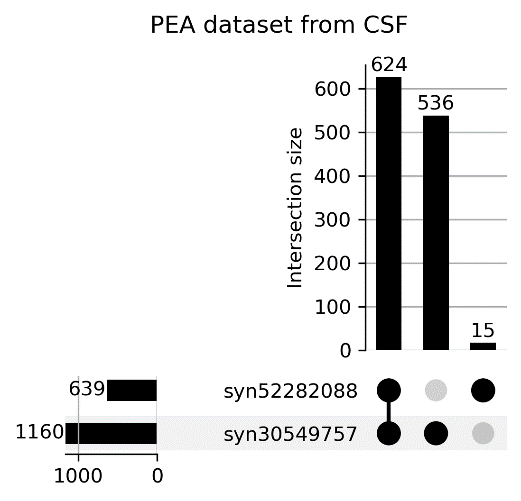
**

**Supplementary Figure 4.** The number of overlapping proteins captured in the different studies of the PEA dataset from the CSF.

**Alt text:** An UpSet plot showing the intersections of proteins across multiple PEA CSF studies.

| Demographic characteristics of the TMT dataset from brain tissue | | | | | | | | | | | | | | | | |
| --- | --- | --- | --- | --- | --- | --- | --- | --- | --- | --- | --- | --- | --- | --- | --- | --- |
|  | Banner | | MSBB | | Rosmap_round1 | | Rosmap_round2 | | Rosmap_CognitiveResilience | | Emory BA9 | | Emory BA24 | | Higginbotham | |
| SynID | syn7170616 | | syn3159438 | | syn3219045 | | syn3219045 | | syn22695346 | |  | |  | |  | |
|  | Control  (n=26) | AD  (n=92) | Control  (n=61) | AD  (n=125) | Control  (n=84) | AD  (n=108) | Control  (n=57) | AD  (n=81) | Control  (n=127) | AD  (n=80) | Control  (n=10) | AD  (n=20) | Control  (n=10) | AD  (n=20) | Control  (n=10) | AD  (n=10) |
| Age, years (Mean ± SD) | 83.4  ±6.3 | 82.7  ±6.1 | 80.9  ±8.3 | 83.9  ±7.2 | 85.2  ±5.0 | 88.2  ±3.0 | 86.8  ±3.9 | 88.6  ±2.6 | 87.4  ±4.4 | 88.0  ±3.2 | 63.7  ±10.2 | 68.3  ±8.4 | 63.0  ±10.3 | 68.2  ±8.3 | 63.7  ±10.2 | 65.1  ±7.4 |
| Sex (M) | 19 | 53 | 29 | 41 | 34 | 27 | 18 | 25 | 39 | 18 | 5 | 11 | 5 | 12 | 5 | 5 |
| Number of batches | 22 | | 19 | | 50 | | 14 | | 26 | | 5 | | 5 | | 5 | |
| Total number of proteins in at least in one sample | 11518 | | 12147 | | 8817 | | 10689 | | 7788 | | 5787 | | 7487 | | 12943 | |
| Number of proteins present in more than 50% of samples | 8615 | | 12147 | | 8793 | | 8144 | | 7766 | | 5787 | | 7487 | | 9019 | |
| Number of proteins shared across all samples of all cohorts | 3870 | | | | | | | | | | | | | | | |
| Total number of missing values across all samples^*^ | 2199 (0.5%) | | 0 (0%) | | 5385 (0.7%) | | 3416 (0.6%) | | 7525 (0.9%) | | 9489 (8.2%) | | 906 (0.8%) | | 1105 (1.4%) | |
| Data sources | AD Knowledge Portal database | | | | | | | | | | [1] | | | | [2] | |
| Reference | [3] | | [4] | | [5-7] | | | | | |  |  |  |  |  |  |

| Demographic characteristics of LFQ dataset from brain tissue | | | | | | | | | | | | | | | | | |
| --- | --- | --- | --- | --- | --- | --- | --- | --- | --- | --- | --- | --- | --- | --- | --- | --- | --- |
|  | ACT | | Banner | | BLSA | | Emory_ADRC | | MSBB | | MayoRNAseq | | UPenn | | UPennPilot | Aging | Umoh |
| SynID | syn5759376 | | syn7170616 | | syn3606086 | | syn3218563 | | syn3159438 | | syn5550404 | | Syn17009177 | | syn5477237 |  |  |
|  | Control  (n=11) | AD  (n=39) | Control  (n=26) | AD  (n=92) | Control  (n=13) | AD  (n=20) | Control  (n=8) | AD  (n=8) | Control  (n=73) | AD  (n=145) | Control  (n=26) | AD  (n=52) | Control (n=47) | AD (n=49) | Control  (n=8) | Control  (n=93) | Control (n=10) |
| Age, years (Mean ± SD) | 85.1  ±4.6 | 87.8  ±3.2 | 83.4  ±6.3 | 82.7  ±6.1 | 80.1  ±9.2 | 84.7  ±7.2 | 65.7  ±6.5 | 64.5  ±5.8 | 80.4  ±8.4 | 83.5  ±7.0 | 89.1  ±1.2 | 83.3  ±6.8 | 65.3  ±8.8 | 78.1  ±10.7 | 71.2  ±14.4 | 46.7  ±9.3 | 72.0  ±18.3 |
| Sex (M,%) | 5 | 16 | 19 | 53 | 10 | 10 | 5 | 4 | 32 | 44 | 10 | 20 | 28 | 22 | 5 | 59 | 5 |
| Number of batches | NaN | | 4 | | NaN | | NaN | | 7 | | 5 | | 10 | | NaN | 3 | NaN |
| Total number of proteins in all samples | 5091 | | 5711 | | 4744 | | 4356 | | 6242 | | 6580 | | 2928 | | 4370 | 2775 | 4178 |
| Number of proteins present in more than 50% of samples | 3707 | | 3874 | | 3988 | | 3061 | | 3437 | | 4119 | | 2878 | | 3428 | 2775 | 3094 |
| Number of proteins shared across all samples of all cohorts | 1311 | | | | | | | | | | | | | | | | |
| Total number of missing values across all samples^*^ | 584 (0.9%) | | 617 (0.4%) | | 116 (0.3%) | | 546 (3%) | | 2455 (0.9%) | | 210 (0.2%) | | 3821 (3%) | | 108 (1%) | 3207 (3%) | 354 (3%) |
| Data sources | AD Knowledge Portal database | | | | | | | | | | | | | | | [8] | [9] |
| Reference | [10] | | [3] | | [11] | |  | | [4] | |  | |  | |  |  |  |

| Demographic characteristics of the TMT dataset from CSF | | | | | | | | | | | | | | | | |
| --- | --- | --- | --- | --- | --- | --- | --- | --- | --- | --- | --- | --- | --- | --- | --- | --- |
| SynID | syn44132374 | | syn20821165 cohort 1 | | syn20821165 cohort 4 | | syn20821165 discovery | | syn20933797 cohort 1 | | syn20933797 cohort 2 | | syn30549757  neat | | syn30549757 deplete | |
|  | Control (n=105) | AD (n=98) | Control (n=63) | AD (n=6) | Control (n=18) | AD (n=17) | Control (n=19) | AD (n=16) | Control (n=147) | AD (n=150) | Control (n=63) | AD (n=6) | Control (n=18) | AD (n=18) | Control (n=20) | AD (n=20) |
| Age, years (Mean ± SD) | 64.5  ±7.7 | 68.2  ±9.1 | 63.2  ±6.9 | 74  ±8.0 | 68.6  ±9.1 | 65.4  ±12.2 | 69.2  ±9.2 | 65.3  ±11.9 | 65.0  ±8.1 | 68.1  ±8.2 | 63.2  ±6.9 | 74.0  ±8.0 | 68.5  ±9.2 | 65.2  ±12.0 | 68.9  ±8.9 | 65.6  ±11.6 |
| Sex (M,%) | 39 | 37 | 26 | 5 | 11 | 10 | 10 | 9 | 41 | 70 | 26 | 5 | 11 | 10 | 11 | 12 |
| Number of batches | 16 | | 12 | | 6 | | 5 | | 38 | | 12 | | 3 | | 11 | |
| CSF tTau, pg/mL | 172.5  ±60.4 | 360.1  ±173.5 | 52.5  ±26.6 | 105.1  ±21.4 | 41.8  ±18.3 | 147.9  ±72.0 | 40.3  ±17.6 | 150.0  ±68.6 | 54.5  ±23.9 | 114.8  ±46.8 | 52.5  ±26.6 | 105.1  ±21.4 | 41.9  ±18.3 | 148.5  ±69.9 | 41.1  ±17.5 | 143.5  ±71.3 |
| CSF pTau, pg/mL | 15.3  ±5.3 | 36.4  ±18.7 | 14.2  ±9.1 | 58.9  ±13.4 | 25.4  ±9.0 | 58.4  ±18.6 | 25.2  ±8.7 | 58.5  ±15.7 | 32.1  ±14.4 | 62.8  ±23.9 | 14.2  ±9.1 | 58.9  ±13.4 | 25.4  ±9.0 | 58.7  ±18.1 | 25.3  ±8.5 | 143.5  ±71.3 |
| CSF Aβ42, pg/mL | 1231.2  ±360.8 | 519.3  ±164.2 | 240.5  ±65.3 | 157.6  ±21.5 | 552.0  ±94.9 | 224.9  ±77.1 | 544.2  ±80.5 | 217.8  ±75.4 | 546.7  ±159.4 | 299.8  ±107.7 | 240.5  ±65.3 | 157.6  ±51.5 | 562.1  ±87.5 | 223.8  ±74.9 | 563.3  ±83.1 | 223.4  ±72.8 |
| Total number of proteins in all samples | 2941 | | 1183 | | 1033 | | 3692 | | 533 | | 792 | | 2229 | | 2944 | |
| Number of proteins present in more than 50% of samples | 1878 | | 797 | | 528 | | 2876 | | 532 | | 790 | | 1791 | | 2694 | |
| Number of proteins shared across all samples of all cohorts | 247 | | | | | | | | | | | | | | | |
| Total number of missing values across all samples* | 0 (0%) | | 232 (1.4%) | | 477 (5.5%) | | 14 (0.2%) | | 2780 (3.8%) | | 232 (1.4%) | | 12 (0.1%) | | 24 (0.2%) | |
| Data sources | Synapse database | | | | | | | | | | | | | | | |
| Reference | [12] | | [2] | | | | | | [8] | | | | [13] | | | |

| Demographic characteristics of the PEA dataset from CSF | | | | |
| --- | --- | --- | --- | --- |
| SynID | syn52282088 | | syn30549757 | |
|  | Control (n=190) | AD (n=235) | Control (n=70) | AD (n=18) |
| Age, years (Mean ± SD) | 57.7±7.6 | 65.8±7.7 | 67.8±5.7 | 65.2±12.0 |
| Sex (M,%) | 120 | 139 | 31 | 10 |
| Number of batches | NaN | | NaN | |
| CSF tTau, pg/mL | 209.3±64.6 | 837.5±385.0 | 58.0±30.0 | 148.5±69.9 |
| CSF pTau, pg/mL | 37.3±9.8 | 99.2±35.8 | 31.8±14.6 | 58.7±18.1 |
| CSF Aβ42, pg/mL | 1137.1±155.7 | 599.7±102.1 | 497.3±135.7 | 223.8±74.9 |
| Total number of proteins in all samples | 664 | | 1196 | |
| Number of proteins present in more than 50% of samples | 639 | | 1160 | |
| Number of proteins shared across all samples of all cohorts | 624 | | | |
| Total number of missing values across all samples^*^ | 0 (0%) | | 126 (0.2%) | |
| Data sources | Synapse database | | | |
| Reference | [14] | | [13] | |

**Supplementary Table 1.** The detailed information of datasets from the brain tissue (TMT and LFQ) and CSF (TMT and PEA).

^*^The proportion of missing values is calculated as the total number of missing values divided by the product of the number of proteins shared across all samples in all cohorts and the total number of samples.)


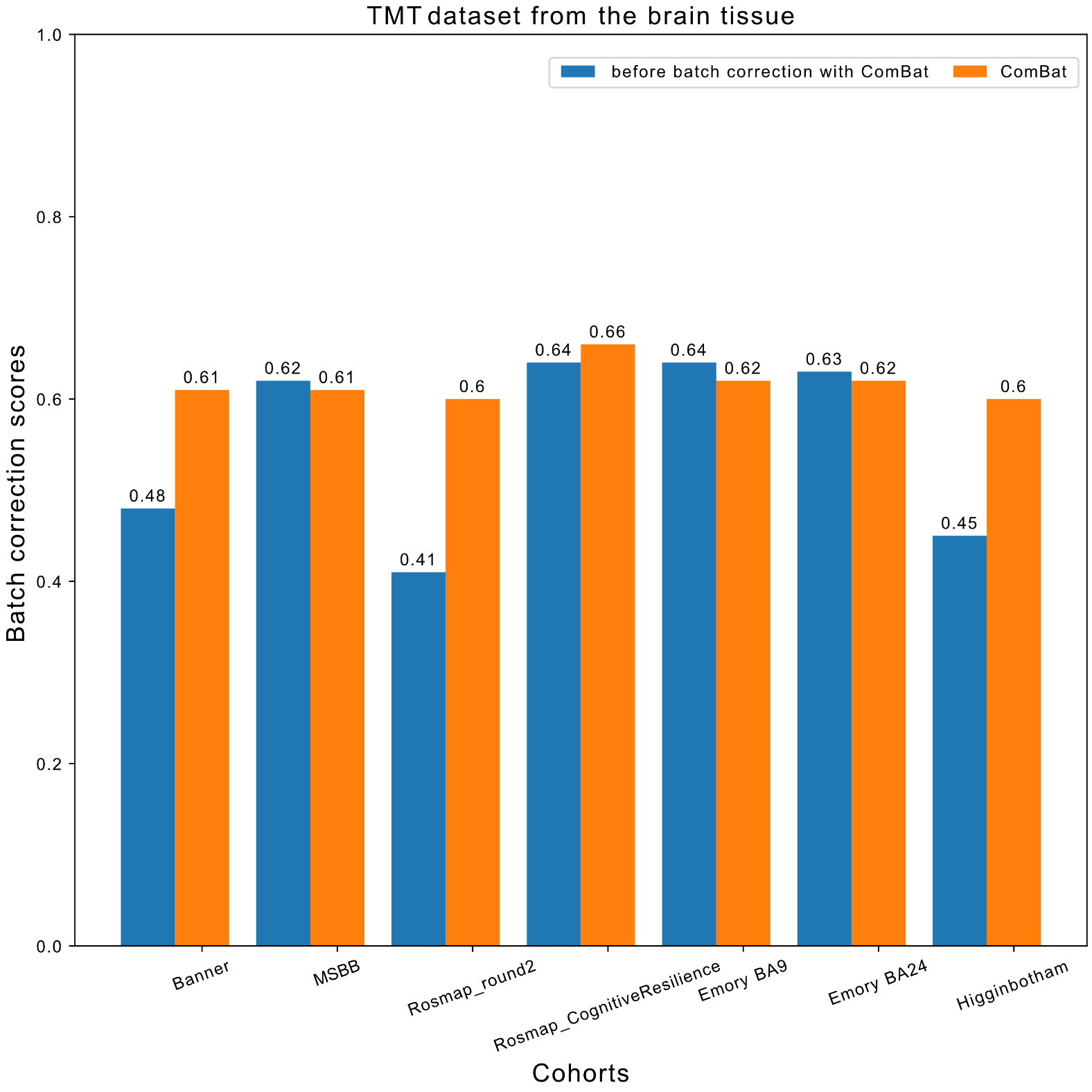


**Supplementary Figure 5.** Batch correction scores across various TMT datasets derived by ComBat from brain tissue.

**Alt text:** A bar plot showing batch correction scores across multiple TMT brain tissue datasets computed using ComBat.

**
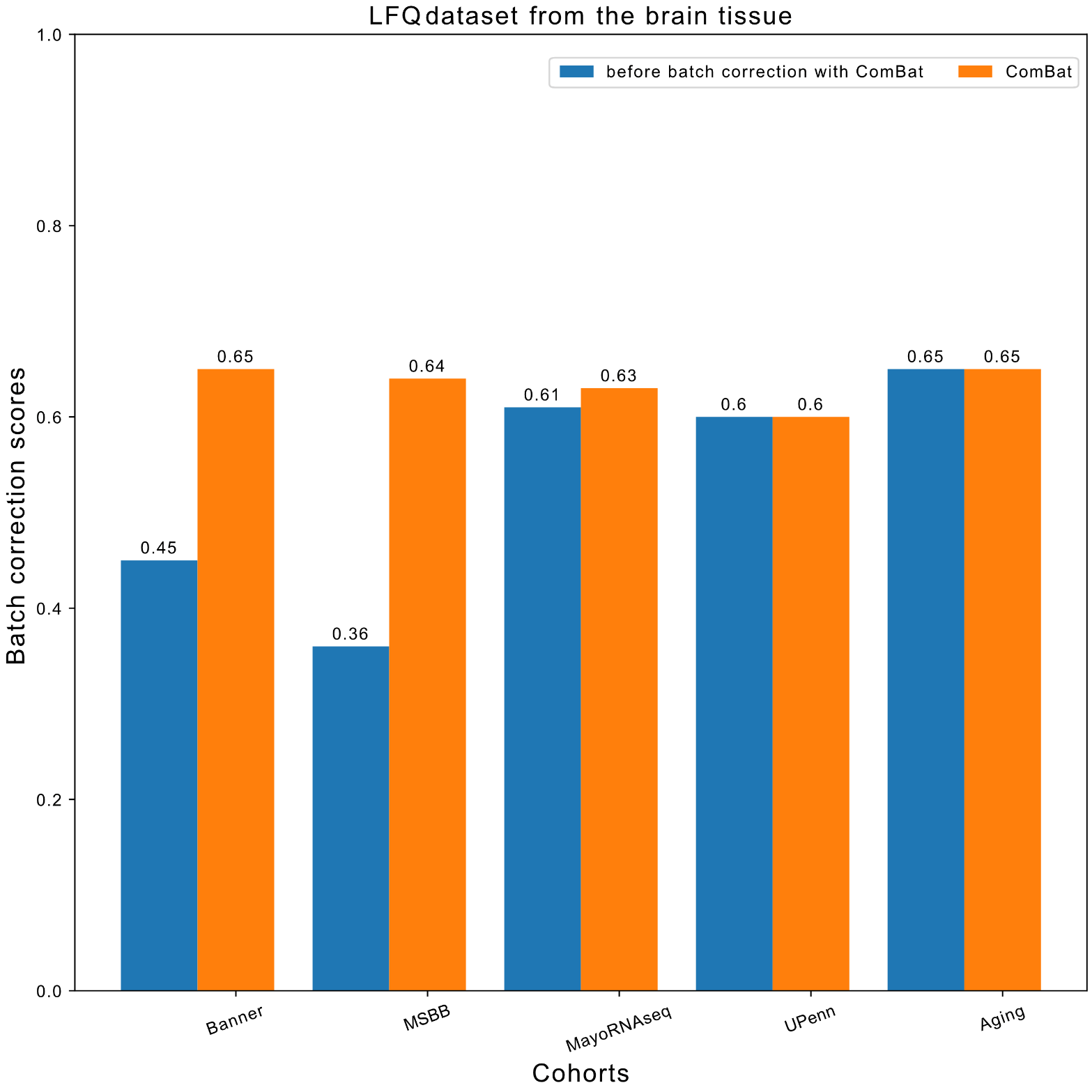
**

**Supplementary Figure 6.** Batch correction scores across various LFQ datasets derived by ComBat from brain tissue.

**Alt text:** A bar plot showing batch correction scores across multiple LFQ brain tissue datasets computed using ComBat.

**
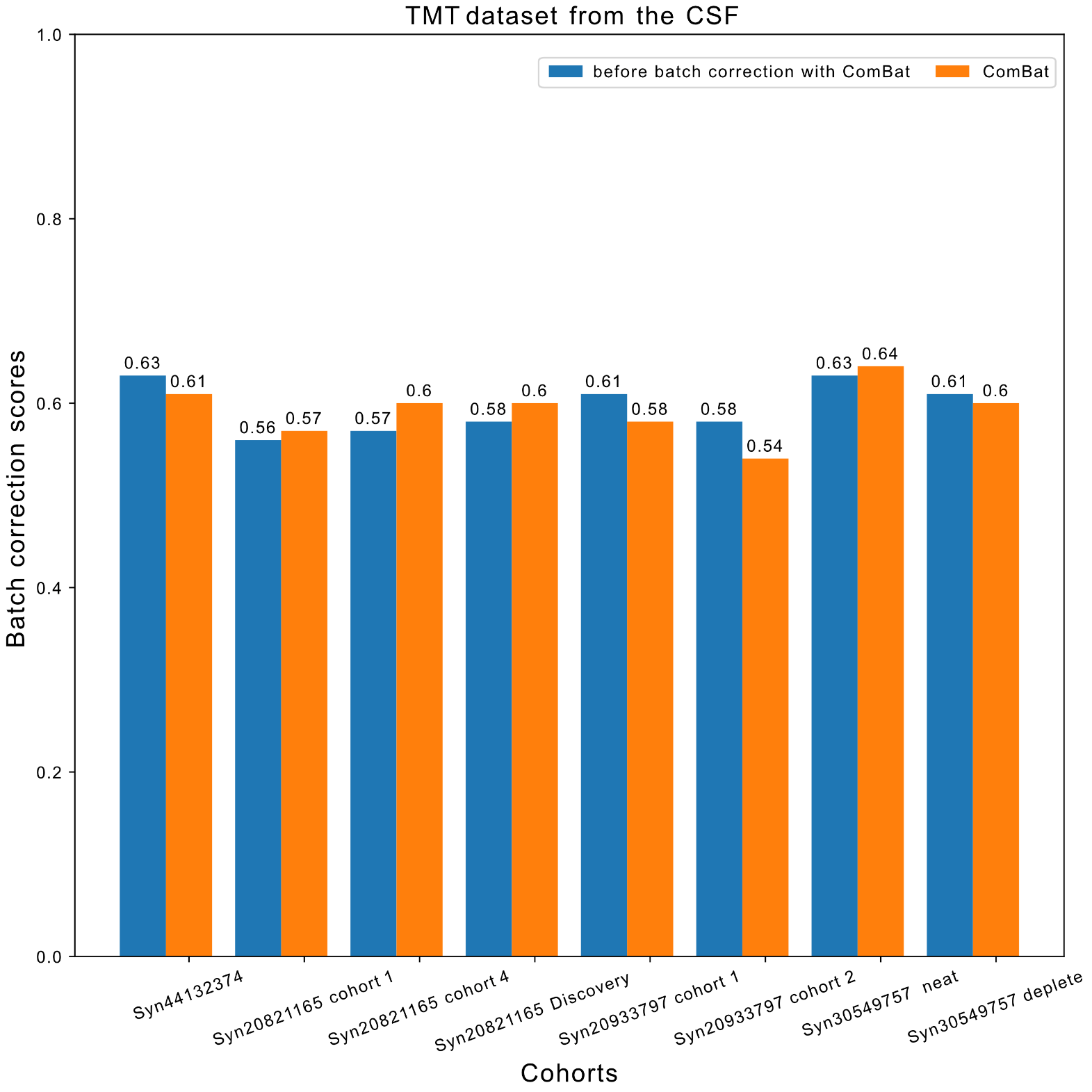
**

**Supplementary Figure 7.** Batch correction scores across various TMT datasets derived by ComBat from CSF.

**Alt text:** A bar plot showing batch correction scores across multiple TMT CSF datasets computed using ComBat.


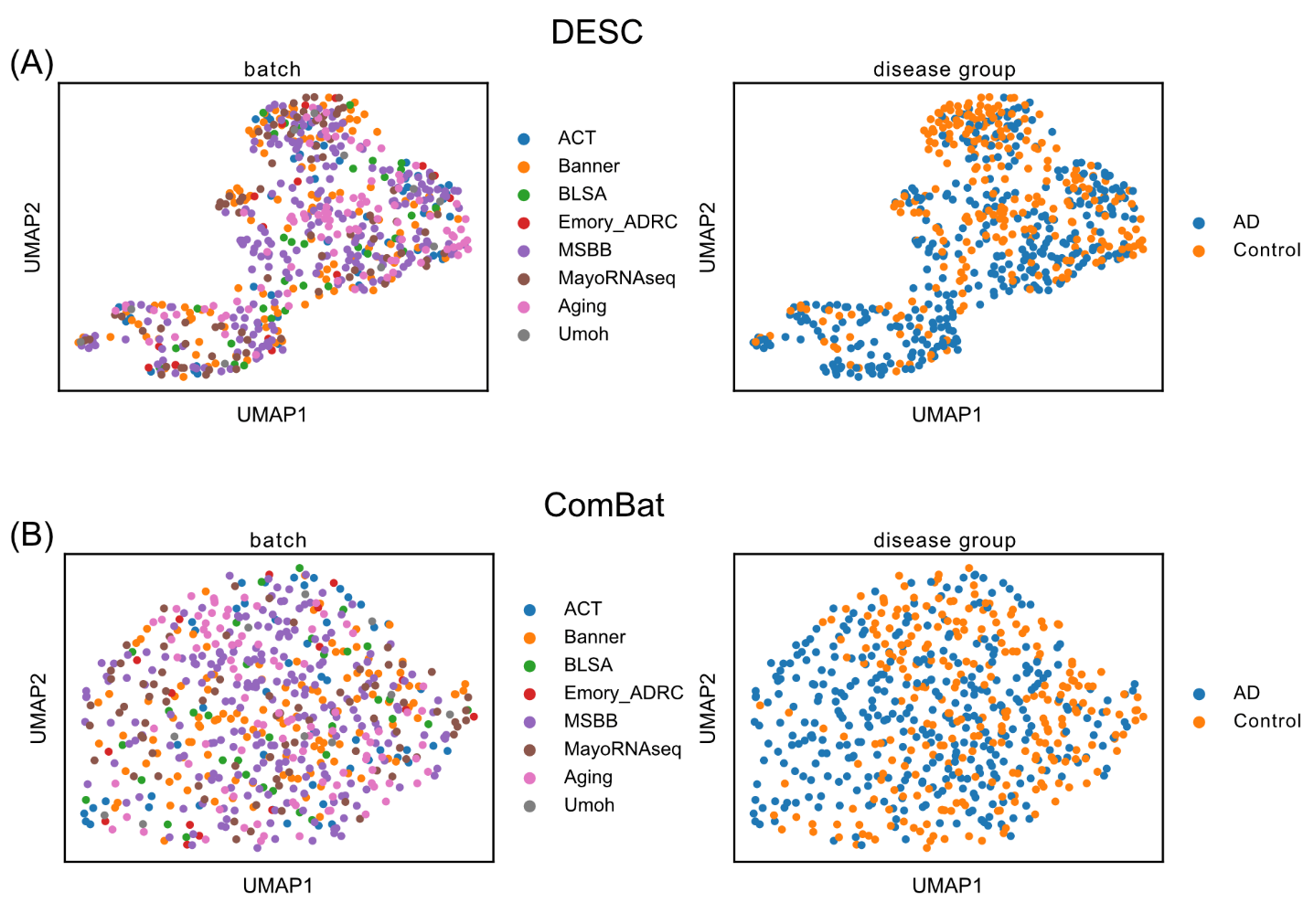


**Supplementary Figure 8.** UMAP visualization of batch effect correction in the LFQ dataset with (A) DESC and (B) ComBat.

**Alt text:** UMAP plots of the LFQ dataset showing batch effect correction using (A) DESC and (B) ComBat.


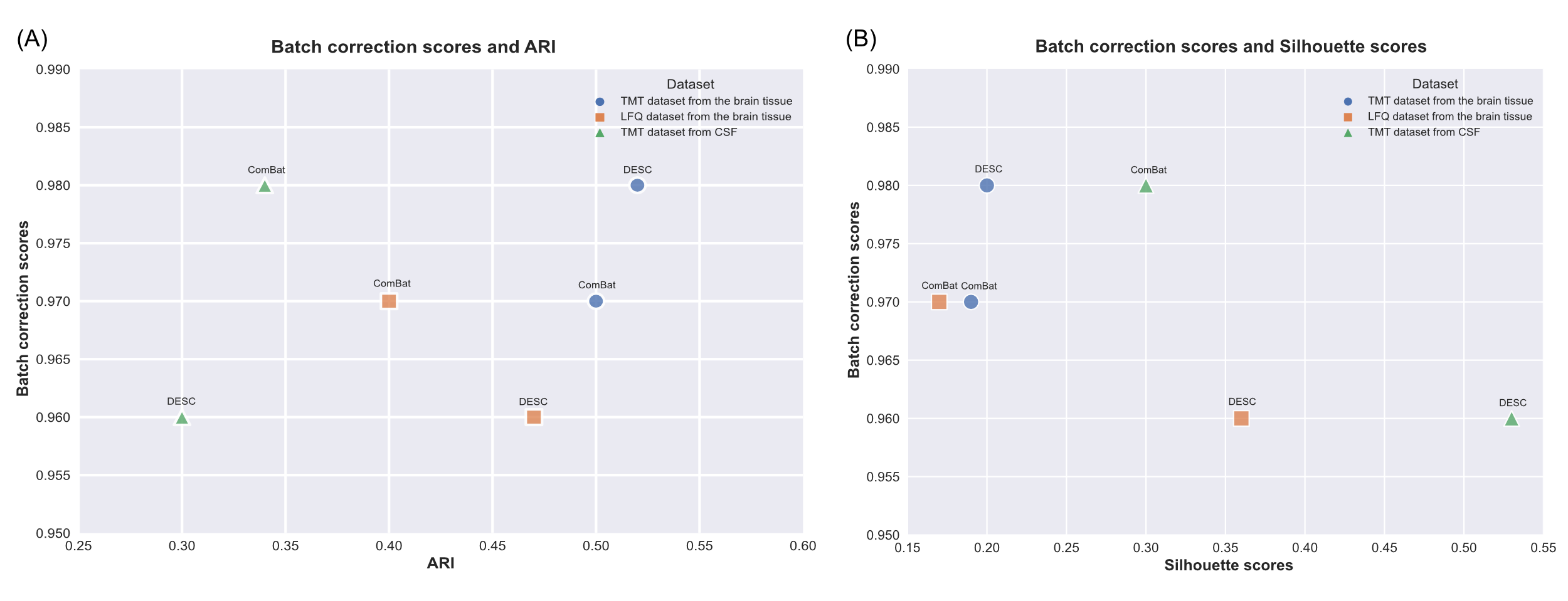


**Supplementary Figure 9.** The scatter plot illustrates the relationship between (A) batch correction scores and ARI and (B) batch correction scores and Silhouette Scores for different datasets and batch correction methods. Each point represents a dataset/method combination. Datasets are distinguished by color and marker shape. The methods, DESC and ComBat, are indicated by text labels adjacent to each point.

**Alt text:** Scatter plots showing the relationship between batch correction scores and (A) ARI, and (B) Silhouette Scores for different datasets and batch correction methods (DESC and ComBat). Points are colored and shaped by dataset, with method indicated by text labels.

| **TMT dataset from the brain tissue** | | | | |
| --- | --- | --- | --- | --- |
| **Genes** | **Uniprot ID** | **Log2FoldChange (AD/Control)** | ***p*-Value** | **Group** |
| AMPD2 | Q01433 | 0.092352 | 8.39E-30 | 1 |
| APP | P05067 | 0.249897 | 1.56E-31 | 1 |
| CSK | P41240 | 0.06881 | 4.53E-30 | 1 |
| CSNK1E | P49674 | 0.059625 | 1.07E-23 | 1 |
| HSPB1 | P04792 | 0.274994 | 3.76E-37 | 3 |
| HTRA1 | Q92743 | 0.23916 | 8.59E-27 | 1 |
| ICAM1 | P05362 | 0.348248 | 8.37E-35 | 3 |
| NTN1 | O95631 | 0.615343 | 1.22E-56 | 3 |
| OLFM1 | Q99784 | -0.14922 | 8.83E-35 | 1 |
| PAFAH1B3 | Q15102 | 0.144492 | 2.14E-42 | 1 |
| PPEF1 | O14829 | -0.19473 | 4.21E-35 | 1 |
| SLC4A10 | Q6U841 | -0.18561 | 3.37E-34 | 1 |
| SPOCK2 | Q92563 | 0.14288 | 2.19E-39 | 1 |
| VGF | O15240 | -0.266214842 | 1.56E-28 | 2 |
| GPNMB | Q14956 | 0.391284994 | 1.74E-28 | 2 |
| GFAP | P14136 | 0.358398029 | 2.44E-27 | 2 |
| C4B | P0C0L5 | 0.287309532 | 1.17E-22 | 2 |
| C4A | P0C0L4 | 0.275362215 | 1.17E-22 | 2 |
| APCS | P02743 | 0.413060847 | 9.35E-26 | 2 |
| OLFML3 | Q9NRN5 | 0.305296723 | 1.64E-14 | 2 |
|  | | | |  |
| **LFQ dataset from the brain tissue** | | | |  |
| VGF | O15240 | -0.60885 | 1.20E-15 | 3 |
| BAG3 | O95817 | 0.280949 | 2.87E-11 | 3 |
| HSPB1 | P04792 | 0.328271 | 9.01E-19 | 3 |
| GFAP | P14136 | 0.343617 | 1.50E-16 | 3 |
| PSMA1 | P25786 | 0.074945 | 1.26E-07 | 1 |
| SNAP25 | P60880 | -0.09628 | 2.24E-08 | 1 |
| NRN1 | Q9NPD7 | -0.23956 | 2.92E-11 | 1 |
| RPH3A | Q9Y2J0 | -0.31858 | 6.07E-14 | 3 |
| PADI2 | Q9Y2J8 | 0.272045 | 1.06E-12 | 3 |
| GFAP | K7EKD1 | 0.561278 | 6.88E-17 | 3 |
| SYT12 | Q8IV01 | -0.29358 | 3.74E-12 | 2 |
| VIM | P08670 | 0.290127142 | 7.21E-08 | 2 |
| PGAM2 | P15259 | 0.304130139 | 3.05E-07 | 2 |

**Supplementary Table 6.** Differential protein abundance between Alzheimer's disease (AD) and Control in the brain tissue using TMT and LFQ datasets. Group 1: selected only by the Random Forest models; Group 2: in the log₂FC top 10 only; Group 3: in the log₂FC top 10 and also selected by the Random Forest models.

| **TMT dataset from the Cerebrospinal fluid** | | | | |
| --- | --- | --- | --- | --- |
| **Genes** | **Uniprot ID** | **Log2FoldChange (AD/Control)** | ***p*-Value** | **Group** |
| CHGB | P05060 | -0.09815 | 1.21E-05 | 1 |
| SPP1 | P10451 | 0.215527 | 2.36E-18 | 3 |
| SCG2 | P13521 | -0.12712 | 9.82E-07 | 1 |
| VGF | O15240 | -0.23801 | 3.90E-10 | 3 |
| NPTXR | O95502 | -0.21528 | 4.77E-11 | 3 |
| CHI3L1 | P36222 | 0.321791 | 8.47E-28 | 3 |
| GAPDH | P04406 | 0.322343 | 2.90E-21 | 3 |
| LDHB | P07195 | 0.197257 | 8.44E-21 | 1 |
| PGK1 | P00558 | 0.286834 | 1.44E-25 | 3 |
| NPTX1 | Q15818 | -0.09047 | 0.000394 | 1 |
| PEBP1 | P30086 | 0.190372 | 1.63E-20 | 1 |
| BASP1 | P80723 | 0.32379 | 1.26E-22 | 3 |
| SOD1 | P00441 | 0.126809 | 8.00E-09 | 1 |
| NPTX2 | P47972 | -0.30493 | 7.08E-24 | 3 |
| PCSK1 | P29120 | -0.17238 | 8.82E-06 | 1 |
| PPIA | P62937 | 0.234613 | 1.28E-21 | 3 |
| NPY | P01303 | -0.27688 | 2.97E-14 | 3 |
|  | | | |  |
| **PEA dataset from the Cerebrospinal fluid** | | | |  |
| SMOC2 | Q9H3U7 | 0.650676 | 2.35E-23 | 1 |
| GLO1 | Q04760 | 0.590111 | 1.38E-29 | 1 |
| MMP10 | P09238 | 0.757394 | 3.84E-37 | 3 |
| ITGB2 | P05107 | 0.8417 | 1.10E-37 | 3 |
| CCN1 | O00622 | 0.703756 | 5.01E-27 | 3 |
| METAP2 | P50579 | 0.30775 | 1.02E-18 | 1 |
| ABL1 | P00519 | 0.570665 | 4.35E-44 | 1 |
| TREM1 | Q9NP99 | 0.512374 | 1.46E-27 | 1 |
| THOP1 | P52888 | 0.55716 | 1.48E-26 | 1 |
| CLEC5A | Q9NY25 | 1.014897 | 4.86E-35 | 3 |
| SDC4 | P31431 | 0.584913 | 5.12E-38 | 1 |
| DDAH1 | O94760 | 0.625824 | 8.46E-34 | 1 |
| TMSB10 | P63313 | 1.005213 | 1.04E-33 | 3 |
| MIF | P14174 | 0.709741 | 1.11E-26 | 3 |
| HSPB1 | P04792 | 0.805435583 | 1.44E-16 | 2 |
| CXCL9 | Q07325 | 0.720264782 | 1.03E-11 | 2 |
| MMP12 | P39900 | 1.066305782 | 7.95E-13 | 2 |
| CHIT1 | Q13231 | 1.158104856 | 6.37E-11 | 2 |

**Supplementary Table 7.** Differential protein abundance between Alzheimer's disease (AD) and Control in Cerebrospinal fluid using TMT and PEA datasets. Group 1: selected only by the Random Forest models; Group 2: in the log₂FC top 10 only; Group 3: in the log₂FC top 10 and also selected by the Random Forest models. The datasets exclude tTau, pTau, and Aβ42.


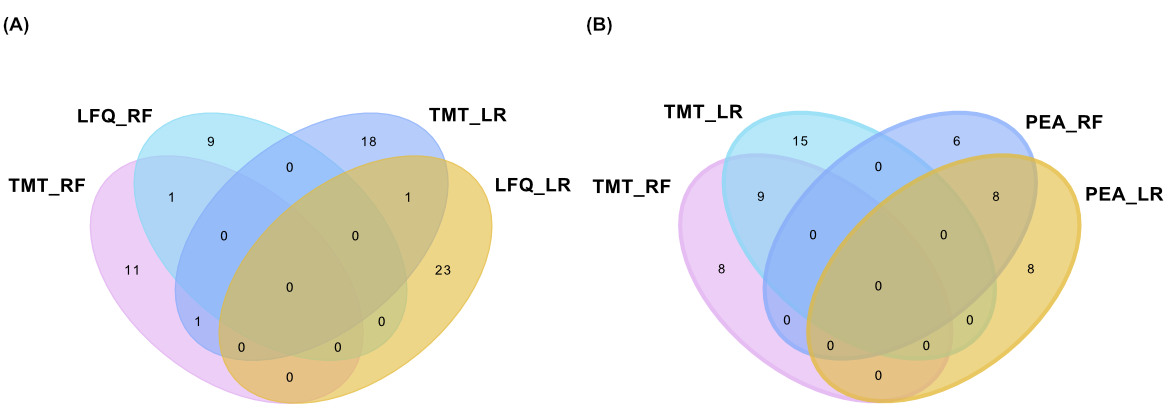


**Supplementary Figure 10.** Overlap between the protein features identified by different machine learning methods and proteomics quantification techniques in (A) brain tissue and (B) cerebrospinal fluid. Abbreviations: TMT, Tandem Mass Tag; LFQ, Label-Free Quantification; PEA, Proximity Extension Assay; RF, Random Forest; LR, Logistic Regression.

**Alt text:** Venn diagrams showing the overlap of protein features identified by different machine learning methods (RF, LR) and proteomics quantification techniques (TMT, LFQ, PEA) in (A) brain tissue and (B) cerebrospinal fluid.

| **Gene name** | **NeuroPro score** | **Proteomics quantification methods** | **Computational analysis** | **Main biological processes** |
| --- | --- | --- | --- | --- |
| GFAP | 31 | LFQ | Random Forest | Neuroinflammation/immune response |
| APP | 28 | TMT | Random Forest | Amyloid pathway |
| HSPB1 | 27 | TMT; LFQ | Random Forest | Neuroinflammation/immune response |
| VGF | 22 | LFQ | Random Forest | Synaptic function/neurotransmission |
| RPH3A | 22 | LFQ | Random Forest | Synaptic function/neurotransmission |
| APOE | 19 | TMT | Logistic Regression | Amyloid pathway |
| PADI2 | 19 | LFQ | Random Forest | Signal transduction/calcium homeostasis |
| STXBP1 | 17 | TMT | Logistic Regression | Synaptic function/neurotransmission |
| HTRA1 | 16 | TMT | Random Forest | Protein folding/Stress response |
| BAG3 | 16 | LFQ | Random Forest | Protein folding/Stress response |
| SNAP25 | 15 | LFQ | Random Forest | Synaptic function/neurotransmission |
| LRPPRC | 14 | LFQ | Logistic Regression | Mitochondrial/Metabolic function |
| OLFM1 | 14 | TMT | Random Forest | Axonal and structural integrity |
| ICAM1 | 13 | TMT | Random Forest | Neuroinflammation/immune response |
| SYT1 | 12 | LFQ | Logistic Regression | Synaptic function/neurotransmission |
| PLCB1 | 12 | LFQ | Logistic Regression |  |
| PAFAH1B3 | 12 | TMT | Random Forest | Lipid metabolism |
| AMPD2 | 11 | TMT | Random Forest | Mitochondrial/Metabolic function |
| GDAP1 | 10 | LFQ | Logistic Regression |  |
| CRIP2 | 10 | LFQ | Logistic Regression | Axonal and structural integrity |
| VPS26A | 10 | LFQ | Logistic Regression |  |
| NTN1 | 10 | TMT | Both Random Forest and Logistic Regression | Synaptic function/neurotransmission |
| NRN1 | 10 | TMT | Random Forest | Synaptic function/neurotransmission |
| SLC4A10 | 10 | TMT | Random Forest | Synaptic function/neurotransmission |
| ATP6V1D | 9 | LFQ | Logistic Regression | Mitochondrial/Metabolic function |
| HLA-DRA | 8 | TMT | Logistic Regression | Neuroinflammation/immune response |
| BDH2 | 8 | LFQ | Logistic Regression | Mitochondrial/Metabolic function |
| RAB27B | 7 | TMT | Logistic Regression |  |
| LRP1 | 7 | LFQ | Logistic Regression | Amyloid pathway |
| KCTD12 | 7 | LFQ | Logistic Regression | Neurodevelopment |
| LANCL2 | 7 | LFQ | Logistic Regression | Lipid metabolism |
| SPOCK2 | 7 | TMT | Random Forest | Axonal and structural integrity |
| PPEF1 | 7 | TMT | Random Forest | Calcium signaling |
| PSMA1 | 7 | LFQ | Random Forest | Protein folding/Stress response |
| IPO5 | 6 | LFQ | Logistic Regression |  |

**Supplementary Table 8.** Proteins computationally selected from brain tissue datasets and present in the NeuroPro database.

| **Gene name** | **Protein name** | **PubMed hits** |
| --- | --- | --- |
| ABCB1 | **ATP-dependent translocase ABCB1** | 115 |
| ACAD9 | Complex I assembly factor ACAD9, mitochondrial | 1 |
| ACOT8 | Acyl-coenzyme A thioesterase 8 | 0 |
| ADGRB1 | Adhesion G protein-coupled receptor B1 | 1 |
| ADGRB3 | Adhesion G protein-coupled receptor B3 | 3 |
| ATAT1 | Alpha-tubulin N-acetyltransferase 1 | 3 |
| CUL5 | Cullin-5 | 4 |
| EIF5 | Eukaryotic translation initiation factor 5 | 3 |
| FIS1 | Mitochondrial fission 1 protein | 57 |
| MGST3 | Glutathione S-transferase 3, mitochondrial | 2 |
| MPDZ | Multiple PDZ domain protein | 3 |
| NCBP2 | Nuclear cap-binding protein subunit 2 | 1 |
| NLGN3 | Neuroligin-3 | 3 |
| PABPC1L2 | Polyadenylate-binding protein 1-like 2 | 0 |
| PDPR | Pyruvate dehydrogenase phosphatase regulatory subunit | 1 |
| PPP1R7 | Protein phosphatase 1 regulatory subunit 7 | 1 |
| PSMB5 | Proteasome subunit beta type-5 | 4 |
| PSME2 | Proteasome activator complex subunit 2 | 1 |
| RAB35 | Ras-related protein Rab-35 | 8 |
| RALB | Ras-related protein Ral-B | 3 |
| RPL35 | Large ribosomal subunit protein uL29 | 2 |
| RPS27 | Small ribosomal subunit protein eS27 | 1 |
| RWDD1 | RWD domain-containing protein 1 | 0 |
| TAB3 | TGF-beta-activated kinase 1 and MAP3K7-binding protein 3 | 0 |
| TALDO1 | Transaldolase | 2 |
| YWHAH | 14-3-3 protein eta | 5 |

**Supplementary Table 9.** Proteins derived from brain tissue and selected by Logistic Regression that were not present in NeuroPro.

**Reference**

1. Johnson ECB, Carter EK, Dammer EB, et al. Large-scale deep multi-layer analysis of Alzheimer’s disease brain reveals strong proteomic disease-related changes not observed at the RNA level. Nature Neuroscience. 2022;25(2):213-25.

2. Higginbotham L PL, Dammer EB, Duong DM, Zhou M, Gearing M, Hurst C, Glass JD, Factor SA, Johnson ECB, Hajjar I, Lah JJ, Levey AI, Seyfried NT. Integrated proteomics reveals brain-based cerebrospinal fluid biomarkers in asymptomatic and symptomatic Alzheimer's disease. Sci Adv. 2020;6(43):eaaz9360.

3. Beach TG, Adler CH, Sue LI, et al. Arizona Study of Aging and Neurodegenerative Disorders and Brain and Body Donation Program. Neuropathology. 2015;35(4):354-89.

4. Wang M, Beckmann ND, Roussos P, et al. The Mount Sinai cohort of large-scale genomic, transcriptomic and proteomic data in Alzheimer's disease. Sci Data. 2018;5:180185.

5. Bennett DA, Buchman AS, Boyle PA, et al. Religious Orders Study and Rush Memory and Aging Project. J Alzheimers Dis. 2018;64(s1):S161-S89.

6. Bennett DA SJ, Arvanitakis Z, Wilson RS. Overview and findings from the religious orders study. Curr Alzheimer Res. 2012;9(6):628-45.

7. Bennett DA SJ, Buchman AS, Barnes LL, Boyle PA, Wilson RS. . Overview and findings from the rush Memory and Aging Project. Current Alzheimer Research. 2012;9(6):646–63.

8. Johnson ECB, Dammer EB, Duong DM, et al. Large-scale proteomic analysis of Alzheimer's disease brain and cerebrospinal fluid reveals early changes in energy metabolism associated with microglia and astrocyte activation. Nat Med. 2020;26(5):769-80.

9. Umoh ME, Dammer EB, Dai J, et al. A proteomic network approach across the ALS-FTD disease spectrum resolves clinical phenotypes and genetic vulnerability in human brain. EMBO Mol Med. 2018;10(1):48-62.

10. Thomas J. Montine JAS, Kathleen S. Montine, Paul K. Crane, Eric B. Larson. Adult Changes in Thought study: dementia is an individually varying convergent syndrome with prevalent clinically silent diseases that may be modified by some commonly used therapeutics. . Curr Alzheimer Res. 2012;9(6):718-23.

11. O'Brien RJ, Resnick SM, Zonderman AB, et al. Neuropathologic Studies of the Baltimore Longitudinal Study of Aging (BLSA). Journal of Alzheimer's Disease. 2009;18(3):665-75.

12. Modeste ES, Ping L, Watson CM, et al. Quantitative proteomics of cerebrospinal fluid from African Americans and Caucasians reveals shared and divergent changes in Alzheimer’s disease. Molecular Neurodegeneration. 2023;18(1).

13. Dammer EB, Ping L, Duong DM, et al. Multi-platform proteomic analysis of Alzheimer's disease cerebrospinal fluid and plasma reveals network biomarkers associated with proteostasis and the matrisome. Alzheimers Res Ther. 2022;14(1):174.

14. Del Campo M, Vermunt L, Peeters CFW, et al. CSF proteome profiling reveals biomarkers to discriminate dementia with Lewy bodies from Alzheimer s disease. Nat Commun. 2023;14(1):5635.
